# Development of fluorinated benzils and bisbenzils as room-temperature phosphorescent molecules

**DOI:** 10.3762/bjoc.16.102

**Published:** 2020-05-29

**Authors:** Shigeyuki Yamada, Takuya Higashida, Yizhou Wang, Masato Morita, Takuya Hosokai, Kaveendra Maduwantha, Kaveenga Rasika Koswattage, Tsutomu Konno

**Affiliations:** 1 Faculty of Molecular Chemistry and Engineering, Kyoto Institute of Technology, Matsugasaki, Sakyo-ku, Kyoto 606-8585, Japan; 2 National Metrology Institute of Japan, National Institute of Advanced Industrial Science and Technology, 1-1-1 Umezono, Tsukuba 305-8560, Japan; 3 Faculty of Technology, Sabaragamuwa University of Sri Lanka, P.O. Box 02, Belihuloya 70140, Sri Lanka

**Keywords:** alkyne oxidation, benzils, bistolanes, fluorinated compounds, phosphorescence

## Abstract

Pure organic phosphorescent molecules are attractive alternatives to transition-metal-complex-based phosphores for biomedical and technological applications owing to their abundance and nontoxicity. This article discloses the design, synthesis, and photophysical properties of fluorinated benzil and bisbenzil derivatives as potential pure organic room-temperature phosphorescent molecules. These compounds were separately converted from the corresponding fluorinated bistolanes via PdCl_2_-catalyzed oxidation by dimethyl sulfoxide, while nonfluorinated bistolane provided the corresponding bisbenzil derivatives exclusively in a similar manner. Intensive investigations of the photophysical properties of the benzil and bisbenzil derivatives in toluene at 25 °C showed both fluorescence with a photoluminescence (PL) band at a maximum wavelength (λ_PL_) of around 400 nm and phosphorescence with a PL band at a λ_PL_ of around 560 nm. Interestingly, intersystem crossing effectively caused fluorinated benzils to emit phosphorescence, which may arise from immediate spin-orbit coupling involving the ^1^(n, π)→^3^(π, π) transition, unlike the case of fluorinated or nonfluorinated bisbenzil analogues. These findings offer a useful guide for developing novel pure organic room-temperature phosphorescent materials.

## Introduction

The development of organic light-emitting molecules is recognized as one of the most important studies because of the broad application of these compounds as fluorescence probes, bio-imaging materials, and biosensors in biomedical diagnostics [1–4] and as organic light-emitting diodes in the technological field [5–8]. Among the organic light-emitting molecules developed thus far, extended π-conjugated compounds (e.g., pyrenes and perylenes) emit fluorescence, which is a radiative deactivation process from the lowest singlet (S_1_) excited state to the ground (S_0_) state [9]. Interestingly, for such π-conjugated molecules, suitable structural modifications can switch the radiative S_1_→S_0_ process to another radiative deactivation process from the triplet (T_1_) excited state to S_0_ via an S_1_→T_1_ intersystem crossing (ISC), resulting in the emission of phosphorescence [9].

Phosphorescent molecules generate two excitons (i.e., 25% S_1_ excitons and 75% T_1_ excitons) by application of an electric field, which is well known for organic light-emitting diodes. S_1_ excitons are converted to T_1_ excitons via an ISC process, finally achieving an excellent light-emitting efficiency (up to 100%) [10]. Therefore, extensive investigations to develop phosphorescent molecules have been performed thus far [11–13].

**Figure 1:**
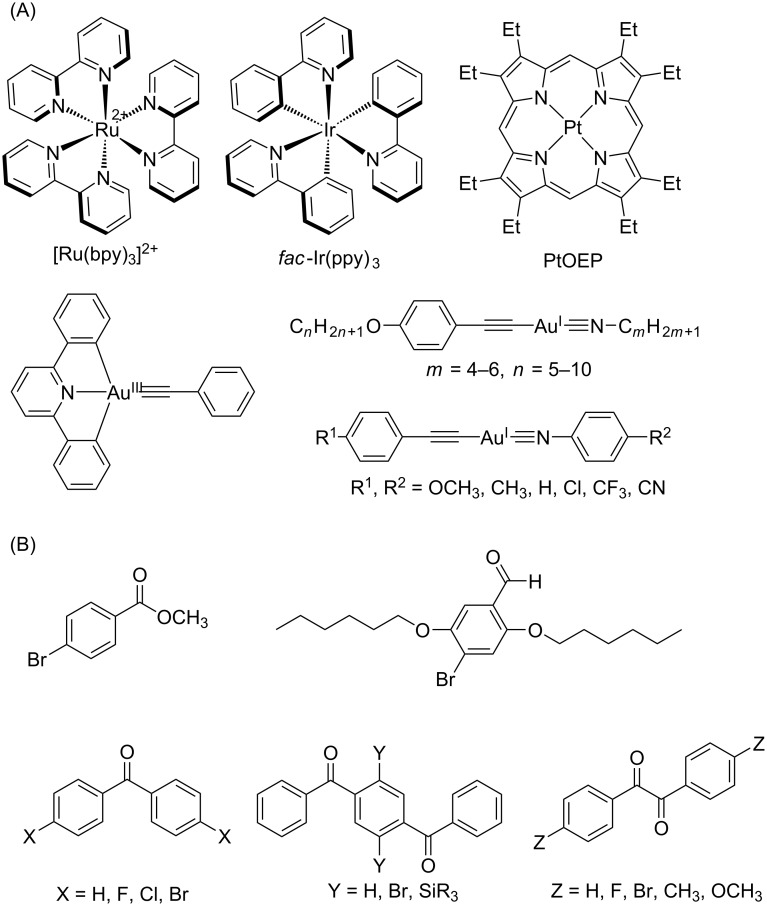
(A) Transition-metal-containing and (B) pure organic phosphorescent materials reported thus far (bpy: 2,2'-bipyridine, ppy: 2-phenylpyridine, OEP: octaethylporphyrin).

It has been established that transition metal complexes containing a heavy atom can promote ISC (e.g., Ru [14], Ir [15–16], Pt [17], and Au [18–21]) (Figure 1A), and this offers a molecular design approach for phosphorescence emission. However, it is becoming necessary to explore alternatives to rare metals because of the latter’s scarcity and toxicity. Owing to recent considerable efforts, several molecular designs have been proposed as alternatives, particularly the use of pure organic phosphorescent molecules [22].

For example, methyl 4-bromobenzoate (Figure 1B) exhibits phosphorescence in the crystalline state via nonradiative ISC to the T_1_ state owing to crystallization-induced restriction of intramolecular motions [23]. Moreover, crystalline 2,5-dihexyloxy-4-bromobenzaldehyde displays green phosphorescence, which stems from rapid ISC due to the heavy atom effect via halogen bonding (C=O···Br) [24]. Moreover, benzophenone- or benzil-type molecules can achieve long-lived phosphorescence owing to a significant acceleration of spin-orbit coupling based on the El-Sayed rule involving the ^1^(n, π)→^3^(π, π) transition [25–26].

**Figure 2:**
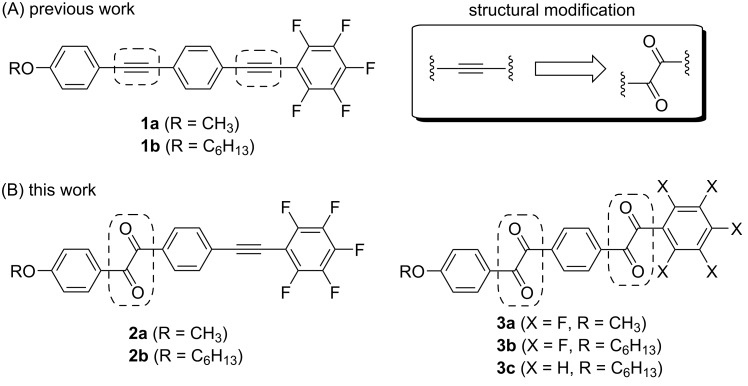
(A) Chemical structures of fluorescent bistolane derivatives previously developed by our group and (B) phosphorescent molecular structures intended for this work.

Over the past few years, our group has intensively investigated fluorinated 1,4-bis(2-phenylethyn-1-yl)benzenes (**1**), a structural class known as bistolanes (Figure 2A), which show prominent fluorescence not only in dilute solution, but also in the crystalline state [27–31].

As a powerful tool to develop novel pure organic phosphorescent molecules, we envisioned the structural modification of the carbon–carbon triple (C≡C) bond in fluorinated bistolane **1** via oxidation to form the corresponding benzil **2** and/or bisbenzil **3** derivatives (Figure 2B). A literature review reveals that bisbenzil-type analogues have not received much attention, despite several publications on benzil-type phosphorescent molecules [25–26,32]. In this study, therefore, we examined the synthesis of novel benzil- and bisbenzil-type molecules via oxidation of fluorinated and nonfluorinated bistolane derivatives and evaluated their photophysical properties in detail.

## Results and Discussion

### Synthesis

**Scheme 1:**
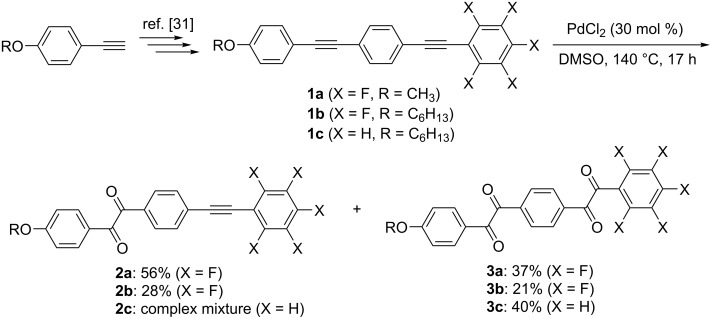
Synthetic pathway for fluorinated benzil (**2**) and bisbenzil (**3**) derivatives.

This study was initiated with the synthesis of fluorinated bisbenzil derivatives. The PdCl_2_-catalyzed oxidation of the C≡C bonds in **1** by dimethyl sulfoxide (DMSO) was performed according to a previously reported procedure (Scheme 1) [33–34].

The methoxy-substituted fluorinated bistolane **1a** was prepared from commercially available 4-ethynylanisole in four facile steps. Interestingly, stirring **1a** in DMSO solution in the presence of 30 mol % of PdCl_2_ at 140 °C for 17 h produced two products (56% yield for the more polar product and 37% yield for the less polar product) after purification with column chromatography. Spectroscopic analyses (i.e., ^1^H, ^19^F, and ^13^C nuclear magnetic resonance (NMR) spectroscopy, infrared spectroscopy, and high-resolution mass spectrometry) successfully identified the more polar product as the half-oxidized benzil **2a** and the less polar one as the fully oxidized bisbenzil **3a**. Fluorinated bistolane **1b**, bearing a hexyloxy chain, also underwent PdCl_2_-catalyzed C≡C oxidation to give rise to the corresponding benzil **2b** and bisbenzil **3b** in 28% and 21% yield, respectively. When nonfluorinated bistolane **1c** was used as the substrate, the corresponding bisbenzil **3c** was obtained in 40% yield as the major product together with an inseparable mixture.

**Scheme 2:**
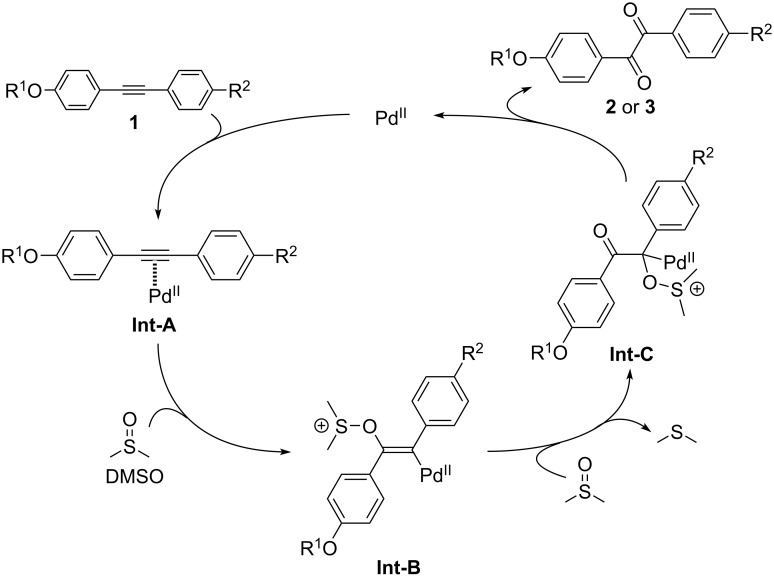
Proposed mechanism of Pd(II)-catalyzed alkyne oxidation by dimethyl sulfoxide (DMSO).

The proposed mechanism of Pd(II)-catalyzed C≡C oxidation is illustrated in Scheme 2 [33]: The catalytic cycle starts with the coordination of the electron-rich C≡C bond to the electron-deficient divalent Pd center, forming the corresponding π-complex (**Int-A**). **Int-A** smoothly undergoes nucleophilic attack by the oxygen atom in DMSO to construct a cationic vinylpalladium(II) species (**Int-B**). Further nucleophilic attack of another DMSO molecule against **Int-B**, followed by elimination of dimethyl sulfide, furnishes a cationic intermediate (**Int-C**). Finally, immediate elimination of dimethyl sulfide and the Pd(II) catalyst gives rise to the corresponding benzil **2**, after which the eliminated Pd(II) catalyst is recycled to provide the oxidation products. The fully oxidized bisbenzil **3** is generated after further Pd(II)-catalyzed C≡C oxidation of the half-oxidized benzil **2** via the same catalytic cycle.

**Figure 3:**
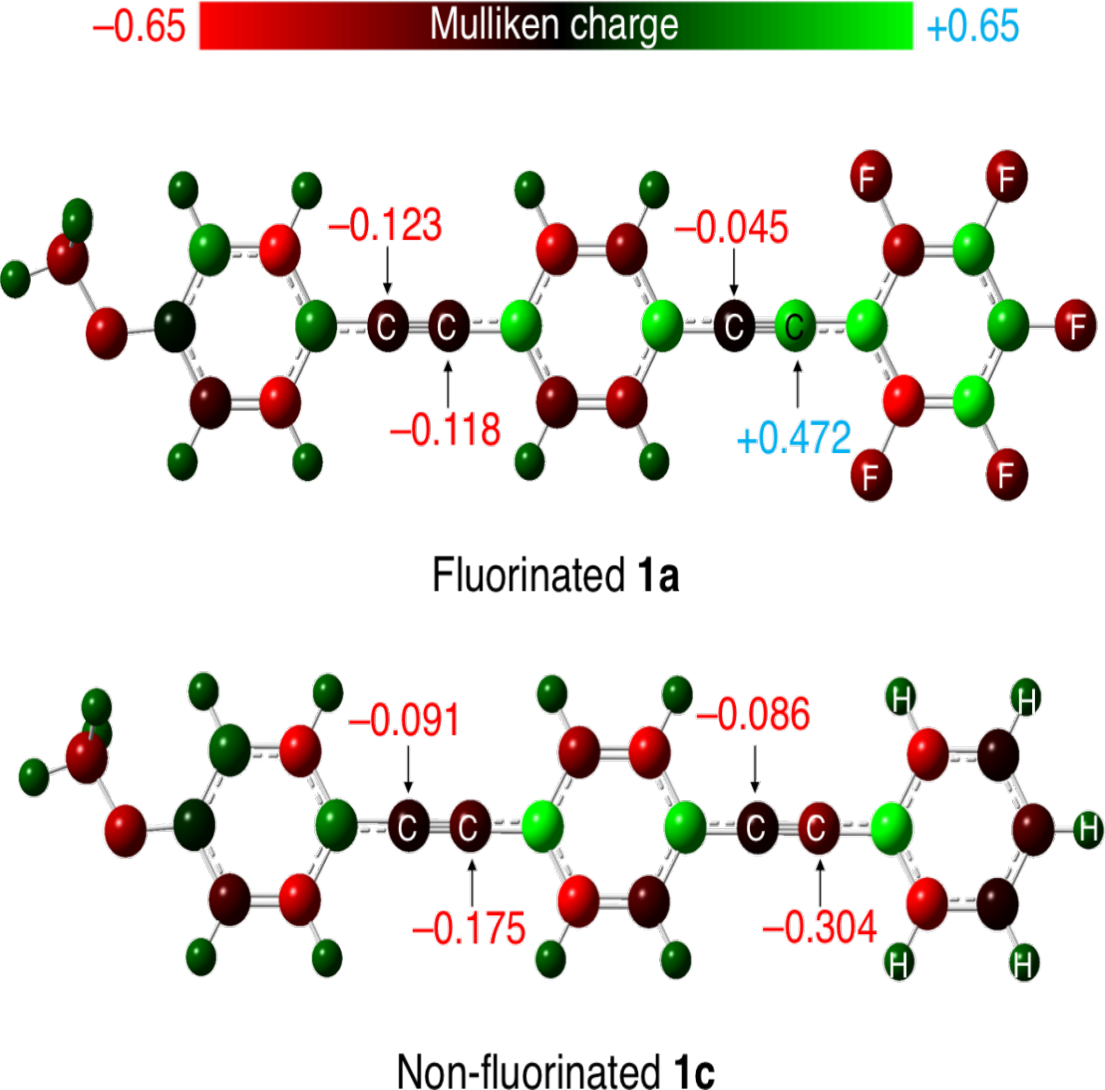
Mulliken charge distributions of fluorinated **1a** and nonfluorinated **1c** obtained from density functional theory calculations [CAM-B3LYP/6-31+G(d) level].

Considering the proposed reaction mechanism, the successful isolation of the half-oxidized benzil derivatives **2a** and **2b** from the oxidation of fluorinated bistolanes **1a** and **1b**, respectively, may be due to the decreased reactivity of the C≡C bond toward Pd(II)-catalyzed C≡C oxidation caused by the adjacent electron-deficient fluorinated aromatic ring. To confirm the electron-withdrawing effect of this fluorinated aromatic ring, the electronic charge at the adjacent C≡C bond was calculated by density functional theory (DFT) using the Gaussian 16 (Revision B.01) software package [35]. As typical examples, the molecular geometries of **1a** and **1c** were optimized at the CAM-B3LYP/6-31+G(d) level of theory. The absence of any imaginary frequency in the vibrational analysis proved that the calculated structures are the minima. Figure 3 shows the calculated Mulliken charge distributions of fluorinated **1a** and nonfluorinated **1c**.

The sp-hybridized carbon adjacent to the fluorinated aromatic ring of **1a** has a significant positive Mulliken charge (+0.472), while that adjacent to the nonfluorinated aromatic ring of **1c** has a negative charge (−0.304). This clearly indicates that the fluorinated aromatic ring retards the Pd(II)-catalyzed oxidation of the adjacent C≡C bond, thereby allowing the isolation of the half-oxidized benzil derivative **2a**. On the basis of this theoretical investigation, the unique reactivities of bistolanes with fluorinated and nonfluorinated aromatic rings toward oxidation by DMSO can be rationalized.

### Photophysical behavior

**Figure 4:**
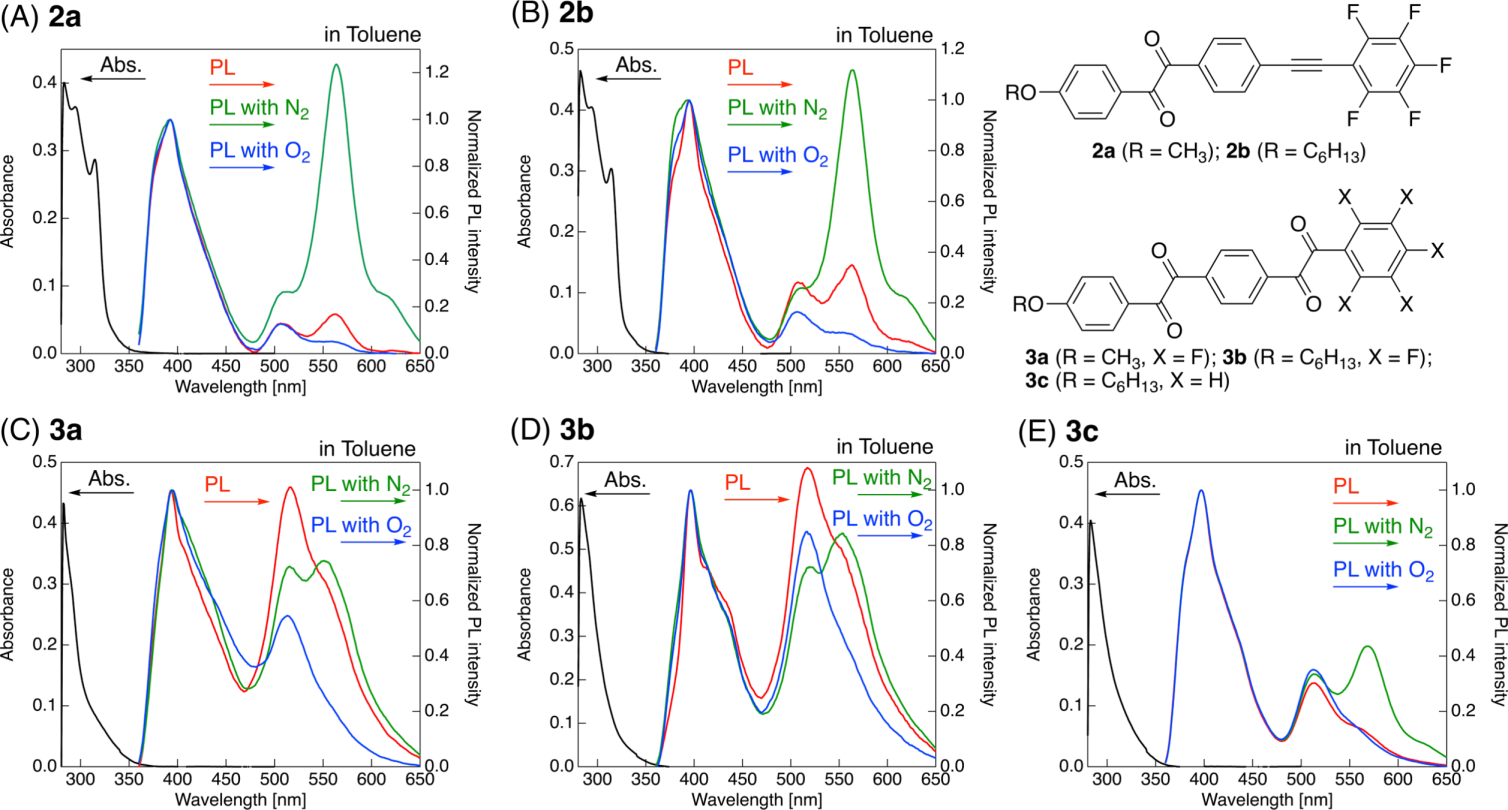
Absorption and photoluminescence (PL) spectra of (A) **2a**, (B) **2b**, (C) **3a**, (D) **3b**, and (E) **3c** in toluene solution. Concentrations: 1.0 × 10^−5^ and 1.0 × 10^−3^ M for absorbance and PL measurements, respectively. Color legend: black: absorption, red: PL as prepared, green: PL under N_2_ atmosphere, and blue: PL under O_2_ atmosphere.

Our interest was then directed toward the photophysical properties of benzil and bisbenzil derivatives, which were freshly purified by column chromatography (eluent: hexane/EtOAc = 5:1 for benzil and 10:1 for bisbenzil) and subsequently recrystallized from hexane. The sample solution concentrations in toluene were 1.0 × 10^−5^ and 1.0 × 10^−3^ M for the absorption and photoluminescence (PL) measurements, respectively, and the absorption and PL spectra are shown in Figure 4. The photophysical data obtained from these measurements are summarized in Table 1.

**Table 1:** Photophysical data from ultraviolet (UV)-visible absorption and steady-state photoluminescence (PL) measurements^a^.

	λ_abs_ [nm] (ε [M^−1^ cm^−1^])	λ_PL_ [nm]^b^ (Φ_PL_)^c^	*I*_560_/*I*_395_		

			pristine	N_2_	O_2_

**2a**	295 (28700), 315 (36500), 407 (160)	395, 406 sh^d^, 507, 563 (0.018)	0.17	1.24	0.05
**2b**	293 (40500), 314 (30400), 407 (180)	395, 406 sh, 507, 563 (0.015)	0.35	1.12	0.08
**3a**	290 (30500), 405 (180)	393, 406 sh, 516, 551 (<0.01)	0.61	0.74	0.19
**3b**	290 (51200), 405 (212)	396, 412, 517, 554 (<0.01)	0.79	0.84	0.50
**3c**	290 (29600), 402 (260)	397, 412, 514, 569 (<0.01)	0.14	0.44	0.14

^a^Toluene solution (concentrations: 1.0 × 10^−5^ and 1.0 × 10^−3^ M for UV-visible absorption and PL measurements, respectively); ^b^Excitation wavelength: 350 nm; ^c^Quantum yield measured using a calibrating sphere. Excitation wavelength: 290 nm. ^d^Shoulder peak.

**Figure 5:**
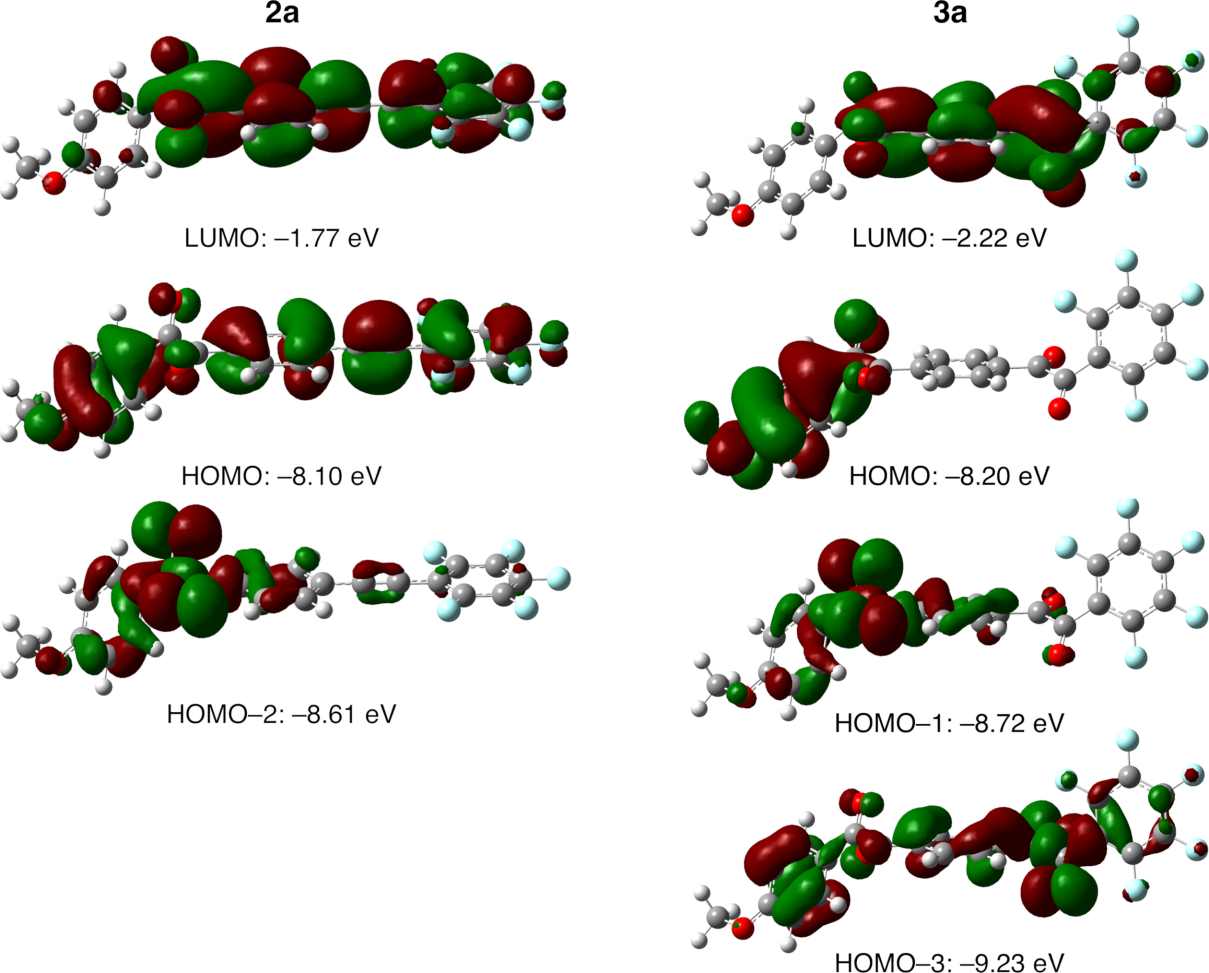
Distributions of molecular orbitals (isosurface value: 0.04 a.u.) involved in vertical electronic transitions in **2a** and **3a** calculated using density functional theory (DFT) and time-dependent DFT at the CAM-B3LYP/6-31+G(d) level (HOMO: highest occupied molecular orbital, LUMO: lowest unoccupied molecular orbital).

The methoxy-substituted fluorinated benzil **2a** in toluene absorbs UV light at 315 and 295 nm with molar extinction coefficients (ε) of 36500 and 28700 M^−1^·cm^−1^, respectively (Figure 4A). Similarly, the toluene solution of the fluorinated benzil with a hexyloxy chain (**2b**) absorbs UV light at 314 (ε: 30400 M^−1^·cm^−1^) and 293 nm (ε: 40500 M^−1^·cm^−1^) (Figure 4B). Both **2a** and **2b** exhibit weak absorption at around 400 nm (ε: ≈170 M^−1^·cm^−1^). As shown in Figure 4C–E, on the other hand, the bisbenzil derivatives **3a**–**c** show an absorption band at 290 nm (ε: 29600–51200 M^−1^·cm^−1^) as the major signal, together with a quite weak absorption band at 402–405 nm (ε: 180–260 M^−1^·cm^−1^). To gain more information about the slight difference between the absorption behaviors of the benzil and bisbenzil derivatives, DFT and time-dependent DFT (TD-DFT) calculations at the CAM-B3LYP/6-31+G(d) level of theory were performed for fluorinated benzil **2a** and bisbenzil derivative **3a** as representative examples. Figure 5 shows the distributions of molecular orbitals involved in vertical electronic transitions in **2a** and **3a**.

The main electronic transition with a relatively large oscillator strength (*f*) in both **2a** and **3a** is the highest occupied molecular orbital (HOMO)→lowest unoccupied molecular orbital (LUMO) transition. Focusing on the orbital distribution, the HOMO lobe of **2a** covers the entire molecule, while the LUMO lobe is localized at the tolane moiety. In the case of **3a**, the HOMO lobe is localized at the methoxy-substituted benzene ring, while the LUMO lobe is localized at the central benzene ring. Accordingly, it can be concluded that the absorption bands at the short-wavelength region (around 290–315 nm) stems from the π–π* transition. The TD-DFT calculation also reveals that n–π* transitions (e.g., HOMO–2→LUMO for **2a** and HOMO–3→LUMO for **3a**) have small values of *f*; thus, the small absorption band at around 400 nm can be safely attributed to an n–π* transition.

Upon irradiation of the toluene solutions of benzil derivatives **2a** and **2b** with a 350 nm UV light, three PL bands at 395, 507, and 563 nm, along with a shoulder signal at around 406 nm, are observed (Figure 4A and 4B). The bisbenzil derivatives, i.e., **3a**–**c**, also show similar PL behavior to the aforementioned benzil analogues: four PL bands with λ_PL_ of 393–397, 406–412, 514–517, and 551–569 nm are observed. To gain more information about the PL process in benzils and bisbenzils, the PL spectra of the toluene solutions (1.0 × 10^−3^ M) were acquired after bubbling with N_2_ or O_2_ gas for 30 min. In general, an O_2_-saturated environment strongly deactivates the triplet states; thus, PL emission stems only from fluorescence. On the other hand, elimination of O_2_ gas from a solution by bubbling with an inert gas (N_2_ or Ar) allows the triplet states to survive for a long lifetime, which possibly leads to a phosphorescence emission. Hence, the elimination of O_2_ gas from solutions by N_2_ gas bubbling (or addition of O_2_ gas in solutions) can judge the presence of phosphorescence, as well as the assignment of PL bands. The obtained PL spectra are superimposed on the PL spectra of a pristine sample (Figure 4). Upon bubbling the solution with N_2_ gas for 30 min, a dramatic enhancement of the PL intensities of benzils **2a** and **2b** at λ_PL_ = 563 nm is observed. The PL intensities of bisbenzils **3a**–**c** at the long-wavelength region between 551 and 569 nm also increase, although the increment rates are not as high as those of **2a** and **2b**. On the contrary, bubbling the toluene solutions of benzil or bisbenzil derivatives with O_2_ gas causes the intensity of the long-wavelength PL band to decrease compared with that of the pristine solution, while the other remaining PL bands in the short-wavelength region do not change. Judging from the PL behavior under N_2_ or O_2_ flow conditions, the PL bands at the short-wavelength region around 395 nm and long-wavelength region around 560 nm can be safely considered fluorescence via radiative deactivation from the S_1_ excited state to the S_0_ state and phosphorescence via electronic transition from the T_1_ excited state to the S_0_ state, respectively. Accordingly, fluorinated benzils and bisbenzils show room-temperature phosphorescence in the solution state.

To understand the effects of structural modification (i.e., the benzil structure with a tolane vs bisbenzil moiety) and incorporation of fluorine atoms on the phosphorescence, the ratio between the peak intensities at ≈395 and ≈560 nm (*I*_560_/*I*_395_) was quantitatively calculated, and the results are summarized in Table 1. The *I*_560_/*I*_395_ values of benzils **2a** and **2b** under N_2_ flow conditions increase up to sevenfold compared with those of the corresponding pristine solutions. On the other hand, the increase in the *I*_560_/*I*_395_ values of fluorinated bisbenzil derivatives **3a** and **3b** is low (only 1.1 times) under N_2_ flow conditions, although the PL intensity of nonfluorinated **3c** increases by approximately three times. Judging from these comprehensive observations, the benzil structure promotes ISC from S_1_ to T_1_, causing increment phosphorescence, unlike the corresponding bisbenzil scaffold. Moreover, fluorine substituents on the bisbenzil molecules cause significant retardation of ISC, leading to a weaker phosphorescence intensity.

Additionally, the quantum yields (Φ_PL_) of the PL bands in the range of 350–600 nm were acquired using an absolute quantum yield measurement system with a calibrated integrating sphere. All samples have a low Φ_PL_ of less than 0.02 (Table 1), meaning that most of the excited states of all samples deactivate nonradiatively. At the moment, we cannot certify the origin of the low Φ_PL_ in terms of the molecular properties, for instance, the main pathway of nonradiative deactivation. Experiments are currently being conducted to better understand the photophysical mechanisms of the excited-state dynamics of these benzil and bisbenzil derivatives.

## Conclusion

In this article, we described the design and synthesis of benzil- or bisbenzil-based room-temperature phosphorescent molecules via a simple oxidation protocol for fluorescent bistolane derivatives. Nonfluorinated bistolane derivatives exclusively gave the corresponding products with a bisbenzil scaffold, whereas the fluorinated bistolane derivatives generated not only mono-oxidized benzil derivatives bearing a fluorinated tolane scaffold, but also the corresponding bis-oxidized bisbenzil derivatives. Based on theoretical calculations, the selective formation of the fluorinated analogues stemmed from the slight modulation of the charge distribution at the alkyne moiety of the reactant induced by the electron-withdrawing fluorine atoms. Evaluation of the photophysical behavior of the benzils and bisbenzils through several PL measurements under N_2_ and O_2_ flow conditions probed the successful room-temperature phosphorescence of the compounds in toluene solution. A fuller understanding of the excited-state dynamics of these benzil and bisbenzil derivatives will assist the development of environmentally benign, pure organic phosphorescent materials.

## Supporting Information

Experimental procedures for the synthesis and characterization of fluorinated benzils **2a** and **2b**, fluorinated bisbenzils **3a** and **3b**, and nonfluorinated bisbenzil **3c**. ^1^H, ^13^C, and ^19^F NMR spectra of **2a**, **2b**, and **3a**–**c**. Cartesian coordinates of the optimized geometries of **1a**, **1c**, **2a**, and **3a** obtained from DFT calculations.

File 1 Experimental preocedures, NMR spectra and Cartesian coordinates.
