# Supplementary material for: Development of fluorinated benzils and bisbenzils as room-temperature phosphorescent molecules
Source: Beilstein J Org Chem. 2020 May 29;16:1154–62. doi: 10.3762/bjoc.16.102 (PMC7277987; doi:10.3762/bjoc.16.102)
Supplement: File 1 — Experimental preocedures, NMR spectra and Cartesian coordinates. [file Beilstein_J_Org_Chem-16-1154-s001.pdf]

BEILSTEIN JOURNAL OF ORGANIC CHEMISTRY

Supporting Information

for

Development of fluorinated benzils and bisbenzils as room-temperature phosphorescent molecules

Shigeyuki Yamada, Takuya Higashida, Yizhou Wang, Masato Morita, Takuya Hosokai, Kaveendra Maduwantha, Kaveenga Rasika Koswattage and Tsutomu Konno

Beilstein J. Org. Chem. **2020,** *16,* 1154–1162. doi:10.3762/bjoc.16.102

Experimental preocedures, NMR spectra and Cartesian coordinates

License and Terms: This is a supporting information file under the terms of the Creative Commons Attribution License (http://creativecommons.org/licenses/by/4.0). Please note that the reuse, redistribution and reproduction in particular requires that the authors and source are credited.
The license is subject to the *Beilstein Journal of Organic Chemistry* terms and conditions: (https://www.beilstein-journals.org/bjoc)

Contents

1. Experimental procedure •••••••••• S2
2. NMR spectra •••••••••• S6
3. Cartesian coordinates •••••••••• S15

1. Experimental procedure

1-1. General

^{1}H and ^{13}C NMR spectra were obtained with a Bruker AVANCE III 400 NMR spectrometer (^{1}H: 400 MHz and ^{13}C: 100 MHz) in chloroform-*d* ($CDCl_3$) solution and the chemical shifts are reported in parts per million (ppm) using the residual proton in the NMR solvent. ^{19}F NMR (376 MHz) spectra were obtained with a Bruker AVANCE III 400 NMR spectrometer in $CDCl_3$ solution with $CFCl_3$ (δ_F = 0 ppm) as an internal standard. Infrared spectra (IR) were recorded in a KBr method with a JASCO FT/IR-4100 type A spectrometer; all spectra were reported in wavenumber (cm^{-1}). High-resolution mass spectra (HRMS) were recorded on a JEOL JMS700MS spectrometer using fast atom bombardment (FAB) methods. All chemicals including solvent were of reagent grade and where necessary were purified in the usual manner prior to use. Column chromatography was carried out on silica gel (Wakogel® 60N, 38–100 μm) and thin-layer chromatography (TLC) analysis was performed on silica gel TLC plates (Merck, Silica gel $60F_{254}$).

1-2. Photophysical properties

UV–vis absorption spectra were recorded on a JASCO V-500 absorption spectrometer. Samples for the absorption measurements were prepared by dissolving the pristine powder solid sample of **2** and **3** in toluene to a concentration of 1.0×10^{-5} M, and the solution was transferred into quartz cuvettes with an optical path length 1.0 cm. The steady-state PL spectra and quantum yields in solution, and pristine powder solid states were acquired using a JASCO FP-6600 fluorescence spectrometer and an absolute PL quantum yield measurement system (Hamamatsu Photonics, C11347-01). A solution-phase sample with a concentration of 1.0×10^{-3} mol L^{-1} was used for PL measurements using quartz cuvettes (1.0 cm path length). The excitation wavelength (λ_{ex}) corresponded to the maximum absorption wavelength.

1-3. Synthesis

Pentafluorinated benzils **2a** and **2b** and bisbenzils **3a** and **3b** were synthesized from the corresponding pentafluorinated bistolanes **1a** and **1b**, which were prepared according to the reported procedure [1] (Scheme S1). The non-fluorinated bisbenzil **3c** was also prepared from the corresponding **1c** in a similar manner.

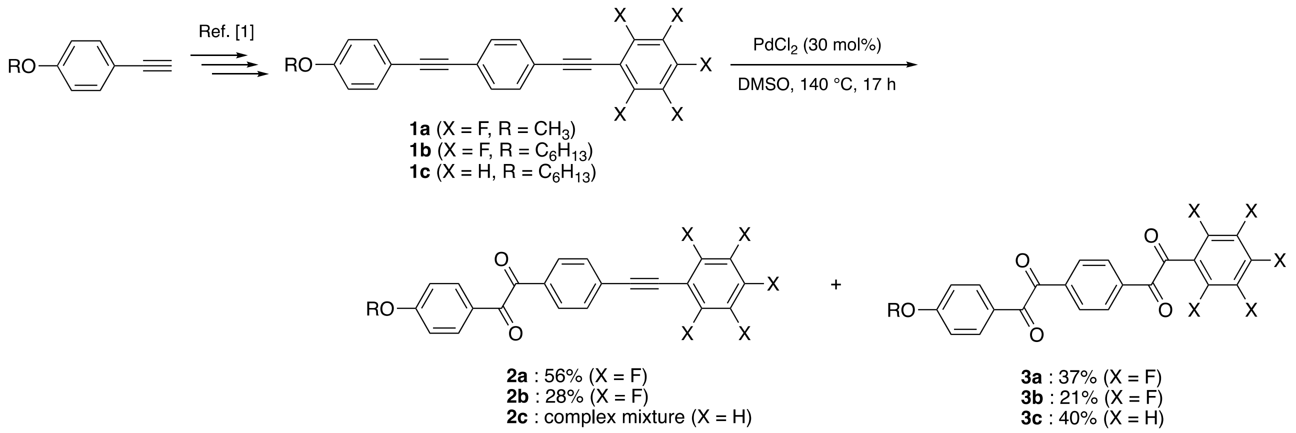

Scheme S1. Synthetic pathway for benzils **2a**, **2b** and bisbenzils **3a**–**c**.

Typical procedure for PdCl$_2$-catalyzed DMSO-oxidation of bistolane 1

In a two-necked round-bottomed flask equipped with a teflon®-coated magnetic stirrer bar was placed pentafluorinated bistolane (**1a**, 0.47 g, 1.2 mmol), freshly prepared according to our previous reports [1], in dimethyl sulfoxide (DMSO, 12 mL). To the solution was added PdCl$_2$ (63 mg, 1.2 mmol) at room temperature, and the whole was heated at 140 °C for 17 h. The resultant was poured into H_2O (30 mL), and extracted with diethyl ether (30 mL, three times). The combined extracts were washed with H_2O (20 mL), brine (20 mL) and dried over anhydrous sodium sulfate (Na_2SO_4), filtered and concentrated under reduced pressure using a rotary evaporator. The residue was purified by silica gel column chromatography (eluent: hexane/EtOAc = 10:1 → 5:1), followed by recrystallization from hexane, providing the corresponding benzil (**2a**, 0.29 g, 0.67 mmol) in 56% yield as a white solid and bisbenil (**3a**, 0.20 g, 0.44 mmol) in 37% yield as a white solid.

1-(4-Methoxyphenyl)-2-[4-{2-(2,3,4,5,6-pentafluorophenyl)ethyn-1-yl}phenyl]-1,2-ethanedione (2a)

Yield: 56% (White solid); Rf = 0.42 (hexane/EtOAc = 5/1); M.p.: 138.2–139.4 °C; ^{1}H NMR (CDCl$_3$): δ 3.90 (s, 3H), 6.99 (d, J = 9.2 Hz, 2H), 7.69 (d, J = 8.8 Hz, 2H), 7.95 (d, J = 9.2 Hz, 2H), 8.00 (d, J = 8.8 Hz, 2H); ^{13}C NMR (CDCl$_3$): δ 55.7, 77.0–77.5 (one carbon was overlapped with $\underline{C}$DCl$_3$), 99.6 (td, J = 18.9, 2.9 Hz), 100.0 (d, J = 2.9 Hz), 114.4, 125.9, 127.7, 129.8, 132.3, 132.5, 133.4, 136.0–139.2 (dm, J = 252.5 Hz), 140.4–143.8 (dm, J = 263.5 Hz), 145.8–148.7 (dm, J = 254.7 Hz), 165.2, 192.4, 193.6; ^{19}F NMR (CDCl$_3$); δ –135.6 to –135.9 (m, 2F), –151.53 (t, J = 20.3 Hz, 1F), –161.6 to –161.9 (m, 2F); IR

[1] (a) Yamada, S.; Miyano, K.; Konno, T.; Agou, T.; Kubota, T.; Hosokai, T. *Org. Biomol. Chem.* **2017**, *15*, 5949; (b) Yamada, S.; Morita, M.; Konno, T. *J. Fluorine Chem.* **2017**, *202*, 54; (c) Yamada, S.; Morita, M.; Agou, T.; Kubota, T.; Ichikawa, T.; Konno, T. *Org. Biomol. Chem.* **2018**, *16*, 5609; (d) Yamada, S.; Miyano, K.; Agou, T.; Kubota, T.; Konno, T. *Crystals* **2019**, *9*, 195; (e) Morita, M.; Yamada, S.; Agou, T.; Kubota, T.; Konno, T. *Appl. Sci.* **2019**, *9*, 1905.

(KBr): ν 3075, 3016, 2971, 2845, 2360, 2345, 2225, 1678, 1600, 1522, 1504, 1266, 1168, 993 cm^{-1}; HRMS (FAB+) *m/z* $[M+H]^+$ calcd for $C_{23}H_{12}F_5O_3$: 431.0707, found 431.0709.

1-(4-Hexyloxyphenyl)-2-[4-{2-(2,3,4,5,6-pentafluorophenyl)ethyn-1-yl}phenyl]]-1,2-ethanedione (2b)

Yield: 28% (Yellow solid); Rf = 0.57 (hexane/EtOAc = 5/1); M.p.: 114.0–114.5 °C; ^{1}H NMR ($CDCl_3$): δ 0.90 (t, *J* = 6.8 Hz, 3H), 1.30–1.38 (m, 4H), 1.46 (quin., *J* = 8.0 Hz, 2H), 1.81 (quin., *J* = 6.8 Hz, 2H), 4.04 (t, *J* = 6.8 Hz, 2H), 6.97 (d, *J* = 8.8 Hz, 2H), 7.69 (d, *J* = 8.8 Hz, 2H), 7.94 (d, *J* = 8.8 Hz, 2H), 7.99 (d, *J* = 8.8 Hz, 2H); ^{13}C NMR ($CDCl_3$): δ 14.0, 22.5, 25.6, 28.9, 31.5, 68.6, 77.0–77.5 (one carbon was overlapped with $\underline{C}DCl_3$)99.6 (td, *J* = 17.6, 2.9 Hz), 100.0 (d, *J* = 2.9 Hz), 114.9, 125.6, 127.7, 129.8, 132.3, 132.4, 133.4, 136.2–139.3 (dm, *J* = 250.1 Hz), 140.4–143.7 (dm, *J* = 262.6 Hz), 145.7–148.8 (dm, *J* = 254.5 Hz), 164.8, 192.4, 193.6; ^{19}F NMR ($CDCl_3$): δ –135.6 to –135.9 (m, 2F), –151.56 (t, *J* = 20.3 Hz, 1F), –161.6 to –161.9 (m, 2F); IR (KBr): ν 3073, 2937, 2857, 2364, 2329, 2217, 1670, 1600, 1522, 1502, 1261, 1170, 992 cm^{-1}; HRMS (FAB+) *m/z* $[M+H]^+$ calcd for $C_{28}H_{22}F_5O_3$: 501.1490, found 501.1491.

1,1'-(1,4-Phenylene)-2-(4-methoxyphenyl)-2'-(2,3,4,5,6-pentafluorophenyl)bis(1,2-ethanedione) (3a)

Yield: 37% (Yellow solid); Rf = 0.42 (hexane/EtOAc = 10/1); M.p.: 113.7–114.8 °C; ^{1}H NMR ($CDCl_3$): δ 3.91 (s, 3H), 7.01 (d, *J* = 8.8 Hz, 2H), 7.97 (d, *J* = 8.8 Hz, 2H), 8.15 (ABq, *J* = 8.8 Hz, 2H), 8.17 (ABq, *J* = 8.8 Hz, 2H); ^{13}C NMR ($CDCl_3$): δ 55.7, 114.5, 125.6, 128.8, 130.3, 130.7, 132.5, 135.0, 136.5–139.8 (dm, *J* = 247.2 Hz), 137.8, 143.2–146.3 (dm, *J* = 271.3 Hz), 144.5–148.0 (dm, *J* = 254.4 Hz), 165.4, 184.3, 187.8, 191.7, 193.3; ^{19}F NMR ($CDCl_3$): δ –137.8 to –138.0 (m, 2F), –143.97 (t, *J* = 20.7 Hz, 1F), –159.4 to –159.7 (m, 2F); IR (KBr): ν 3056, 2980, 2851, 1735, 1700, 1686, 1602, 1498, 1425, 1257, 1172, 1001, 888 cm^{-1}; HRMS (FAB+) *m/z* $[M+H]^+$ calcd for $C_{28}H_{22}F_5O_5$: 533.1388, found 533.1380.

1,1'-(1,4-Phenylene)-2-(4-hexyloxyphenyl)-2'-(2,3,4,5,6-pentafluorophenyl)bis(1,2-ethanedione) (3b)

Yield: 21% (Yellow solid); Rf = 0.34 (hexane/EtOAc = 10/1); M.p.: 94.3–95.9 °C; ^{1}H NMR ($CDCl_3$): δ 0.91 (t, *J* = 6.4 Hz, 3H), 1.30–1.52 (m, 6H), 1.81 (quin., *J* = 6.8 Hz, 2H), 4.05 (t, *J* = 6.8 Hz, 2H), 6.98 (d, *J* = 8.8 Hz, 2H), 7.94 (d, *J* = 8.8 Hz, 2H), 8.14 (ABq, *J* = 8.8 Hz, 2H), 8.17 (ABq, *J* = 8.8 Hz, 2H); ^{13}C NMR ($CDCl_3$): δ 14.0, 22.5, 25.6, 28.9, 31.5, 68.6, 115.0, 125.4, 128.8, 130.3, 130.7, 132.5, 134.9, 136.2–139.3 (dm, J = 250.1 Hz), 137.8, 140.4–143.7 (dm, J = 262.6 Hz), 145.7–148.8 (dm, J = 254.5 Hz), 165.0, 184.4 (d, *J* = 11.1 Hz), 187.8, 191.8, 193.4, three sp^2 carbons attached with fluorine atom cannot be detected due to low solubility; ^{19}F NMR ($CDCl_3$): δ –137.8 to –138.0 (m, 2F), –143.97 (tt, *J* = 20.7, 5.6 Hz, 1F), –159.4 to –159.7 (m, 2F); IR (KBr): ν 3098, 2939, 2857, 1684, 1603, 1498, 1255, 1172, 991, 896 cm^{-1}; HRMS (FAB+) *m/z* $[M+H]^+$ calcd for $C_{28}H_{22}F_5O_3$: 501.1490, found 501.1491.

1,1'-(1,4-Phenylene)-2-(4-hexyloxyphenyl)-2'-phenylbis(1,2-ethanedione) (3c)

Yield: 40% (Yellow solid); Rf = 0.45 (hexane/EtOAc = 5/1); M.p.: 115.6–116.8 °C; ^{1}H NMR ($CDCl_3$): δ 0.90 (t, J = 7.6 Hz, 3H), 1.30–1.37 (m, 4H), 1.46 (quin., J = 8.0 Hz, 2H), 1.80 (quin., J = 7.6 Hz, 2H), 4.04 (t, J = 6.4 Hz, 2H), 6.97 (d, J = 9.2 Hz, 2H), 7.53 (t, J = 8.0 Hz, 2H), 7.68 (tt, J = 7.2, 1.6 Hz, 1H), 7.92 (d, J = 8.8 Hz, 2H), 7.96 (dd, J = 8.4 Hz, 1.2 Hz, 2H), 8.10 (s, 4H); ^{13}C NMR ($CDCl_3$): δ 14.0, 22.5, 25.6, 28.9, 31.4, 68.6, 114.9, 125.3, 129.1, 129.9, 130.16, 130.22, 132.5, 132.6, 135.2, 136.9, 137.4, 164.9, 191.9, 193.3, 193.5, 193.6; IR (KBr): ν 3069, 2951, 2857, 2361, 2332, 1675, 1663, 1601, 1572, 1263, 1210, 1169, 887 cm–1; HRMS (FAB+) *m/z* $[M+H]^+$ calcd for $C_{28}H_{27}O_5$: 443.1859, found 443.1869.

2. NMR spectra

^{1}H NMR spectrum for **2a** ($CDCl_3$, 400 MHz)

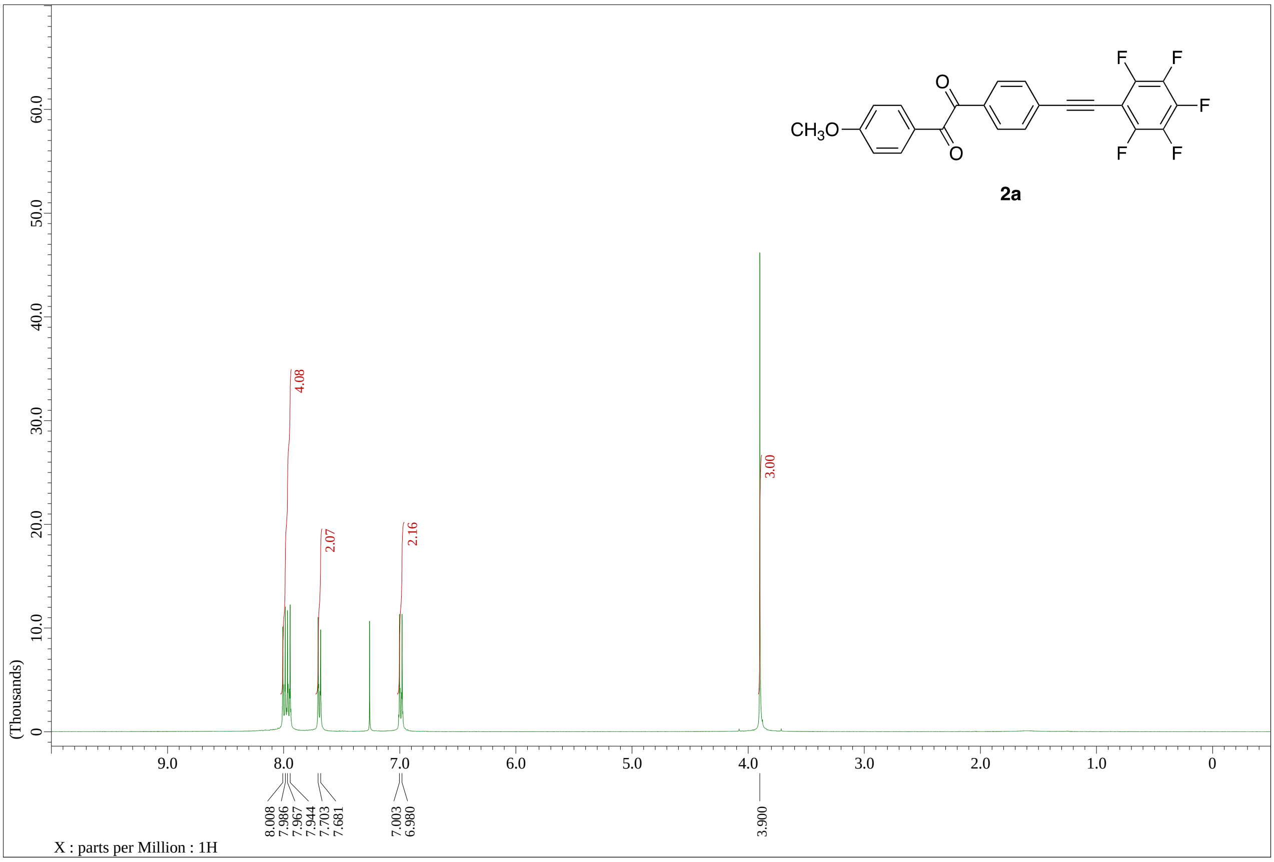

^{13}C NMR spectrum for **2a** ($CDCl_3$, 100 MHz)

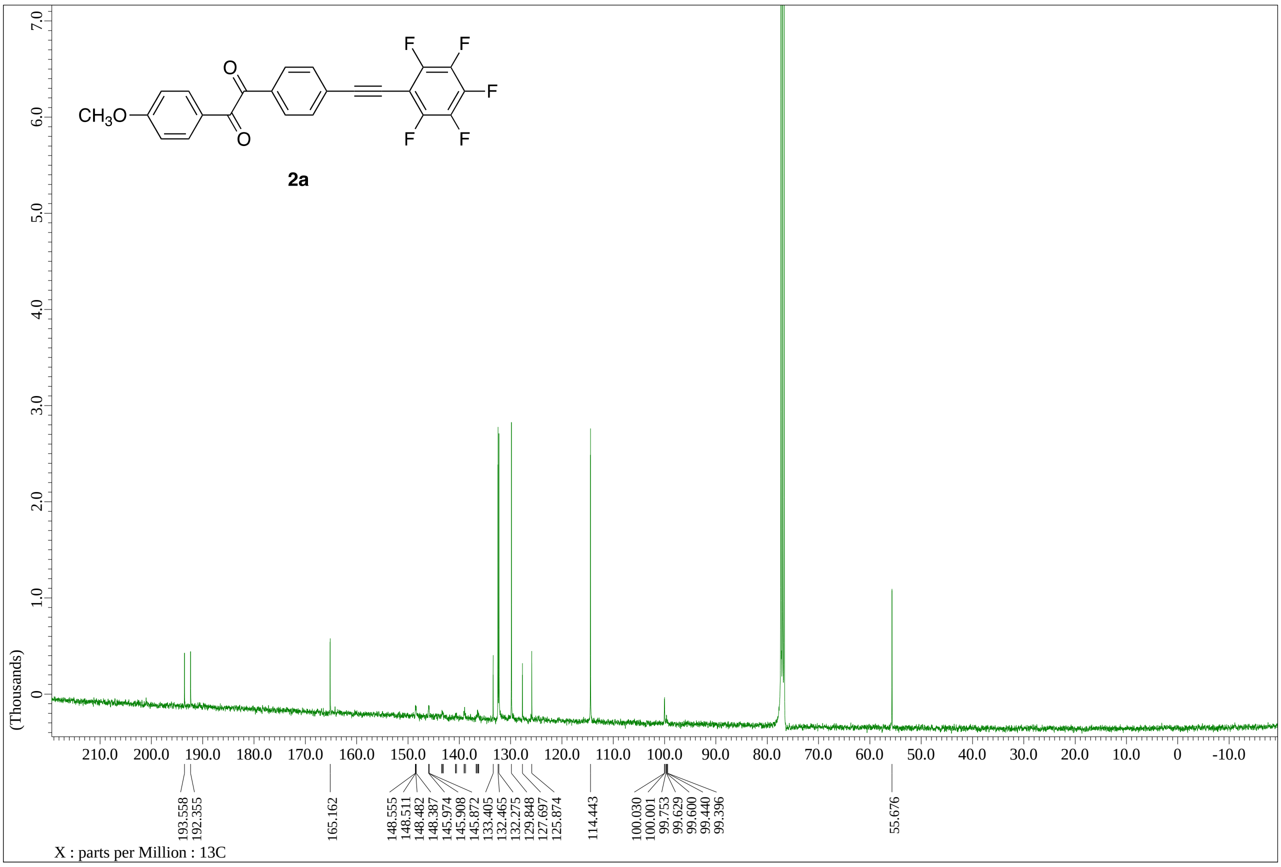

^{19}F NMR spectrum for **2a** ($CDCl_3$, 376 MHz)

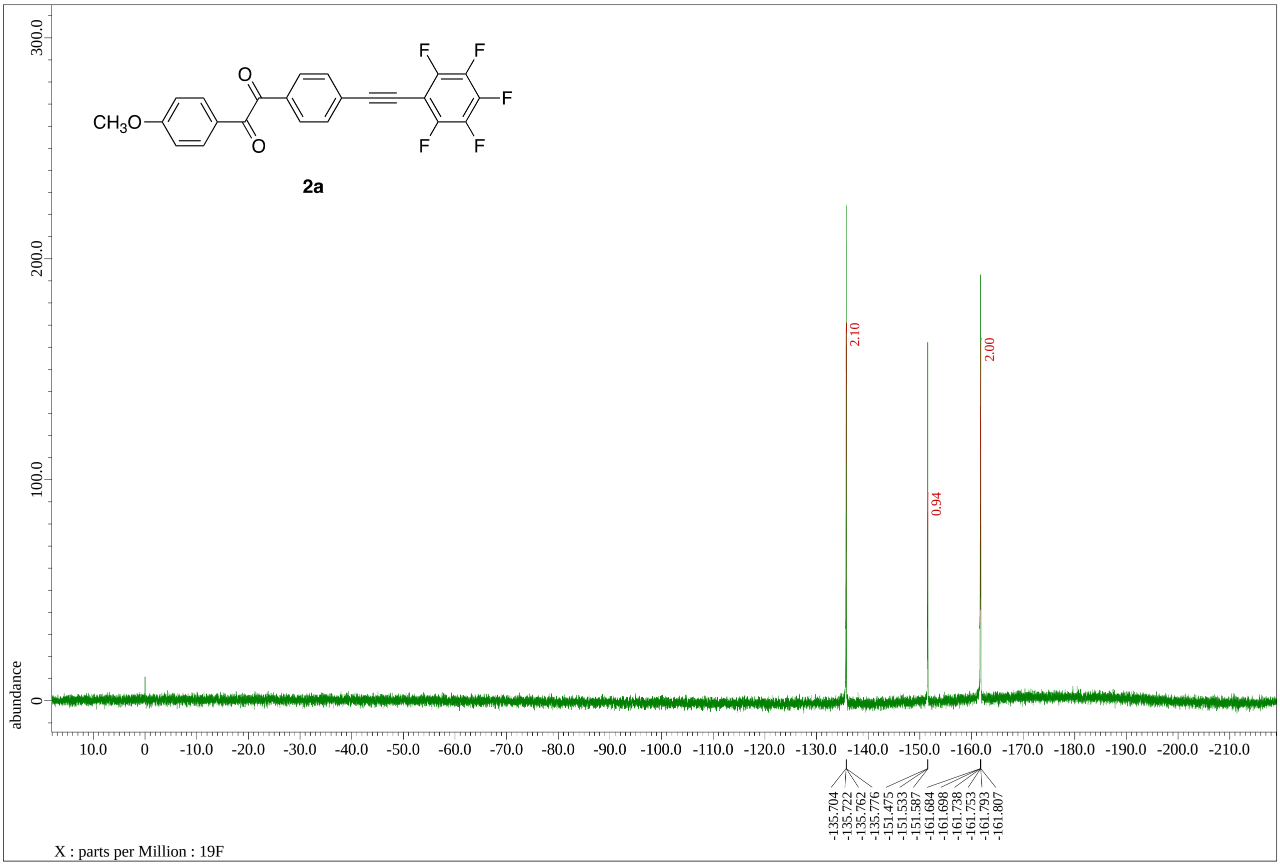

^{1}H NMR spectrum for **2b** ($CDCl_3$, 400 MHz)

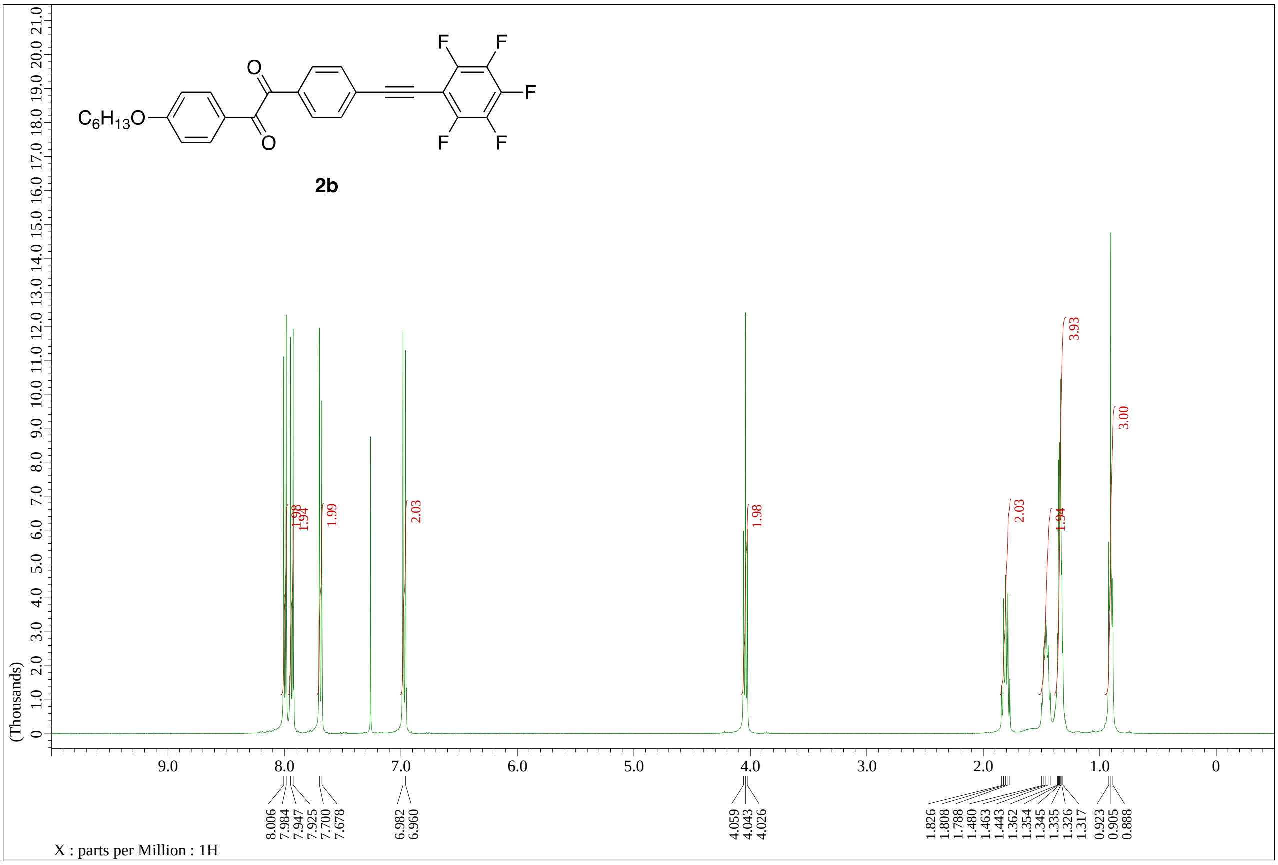

^{13}C NMR spectrum for **2b** ($CDCl_3$, 100 MHz)

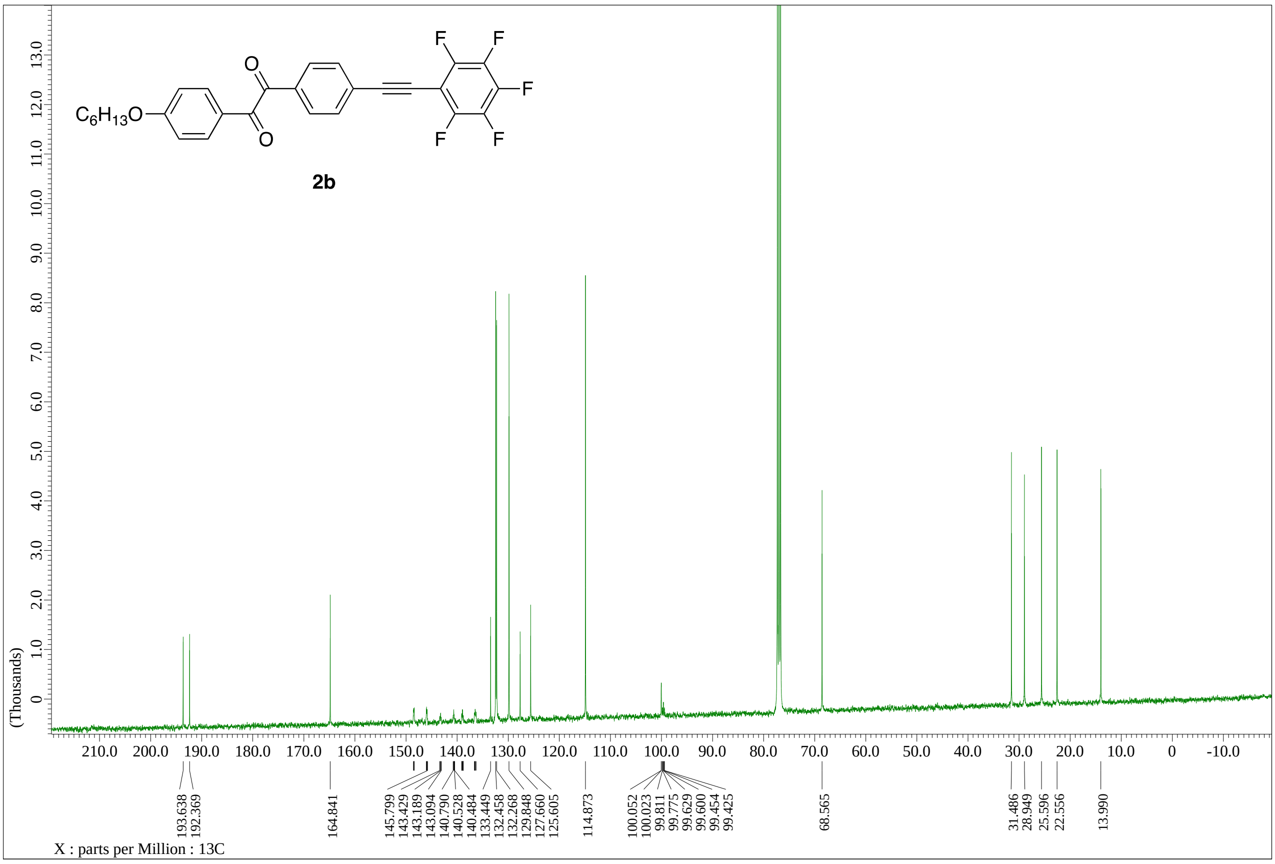

^{19}F NMR spectrum for **2b** ($CDCl_3$, 376 MHz)

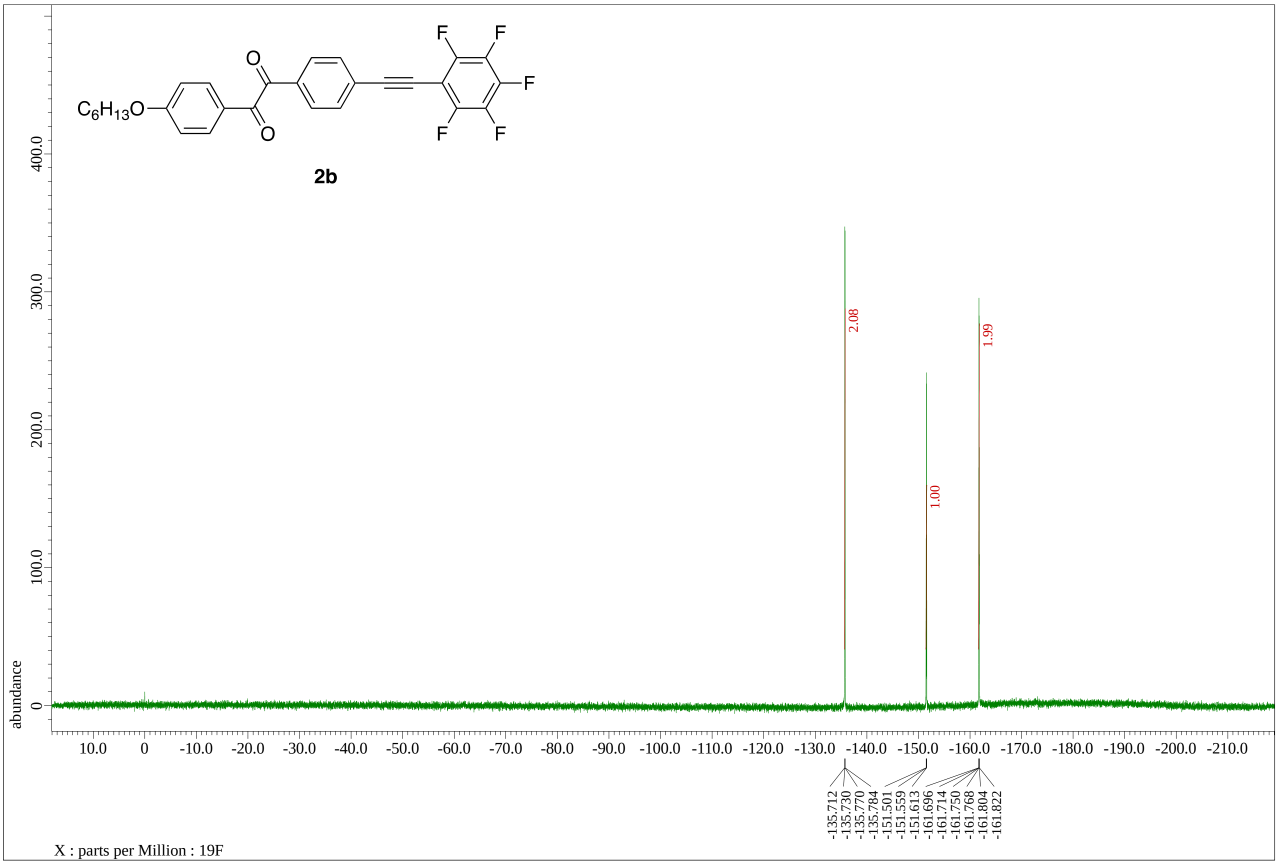

^{1}H NMR spectrum for **3a** ($CDCl_3$, 400 MHz)

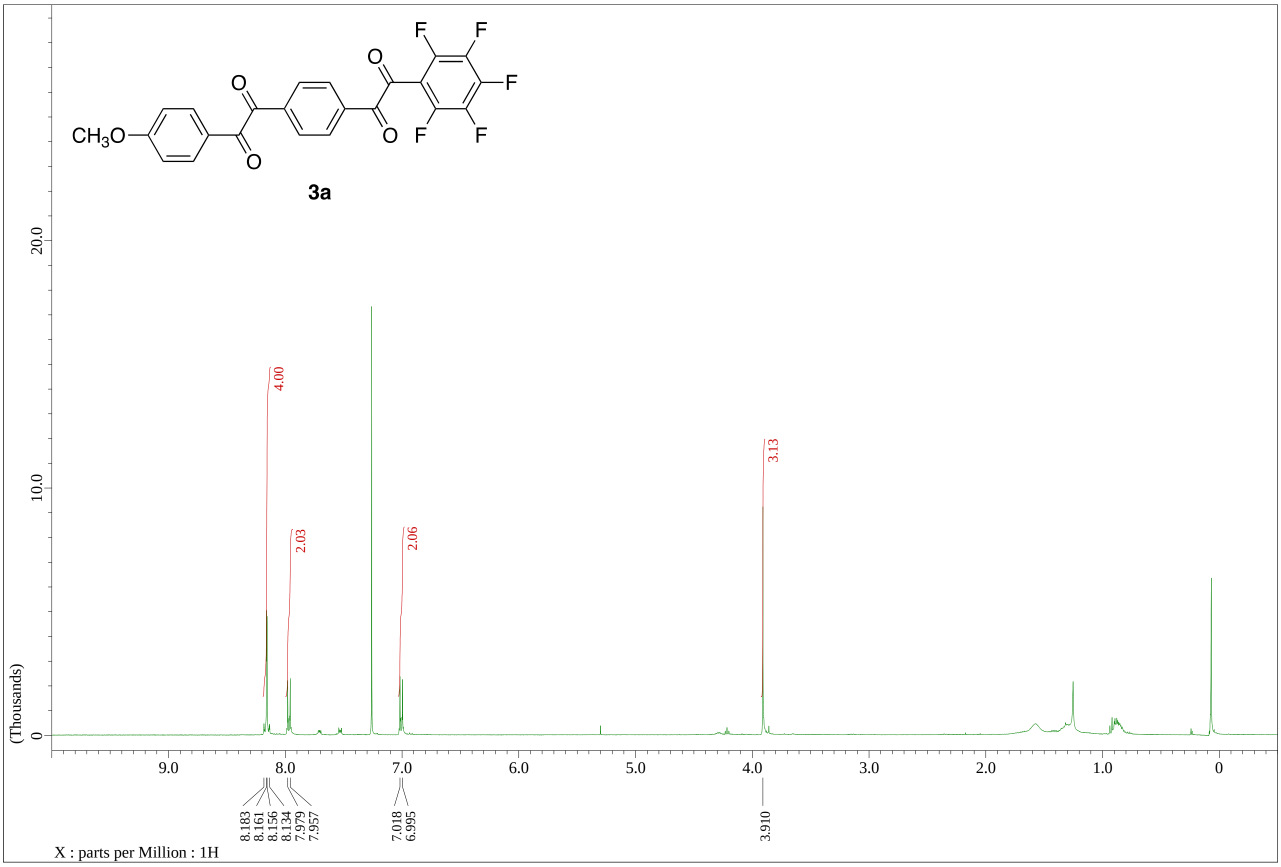

^{13}C NMR spectrum for **3a** ($CDCl_3$, 100 MHz)

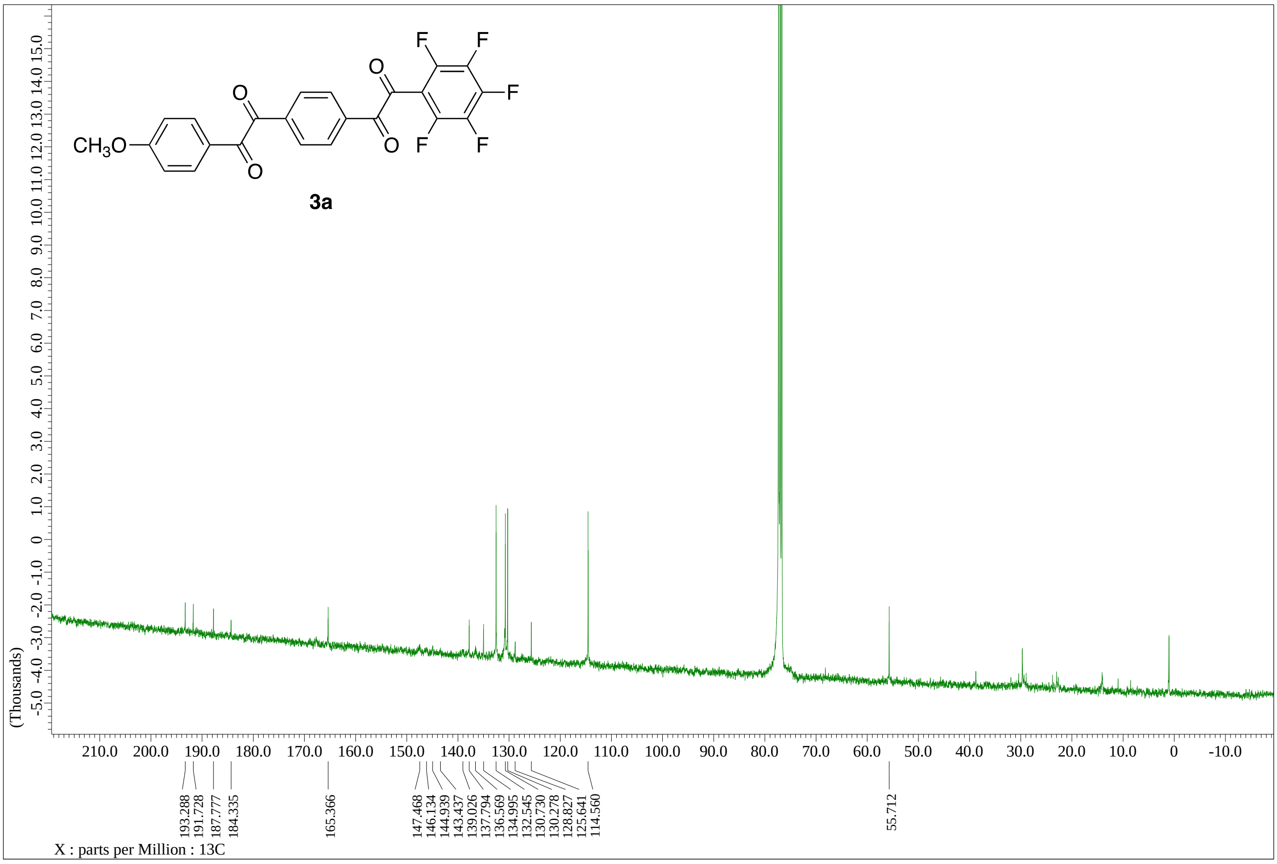

^{19}F NMR spectrum for **3a** ($CDCl_3$, 376 MHz)

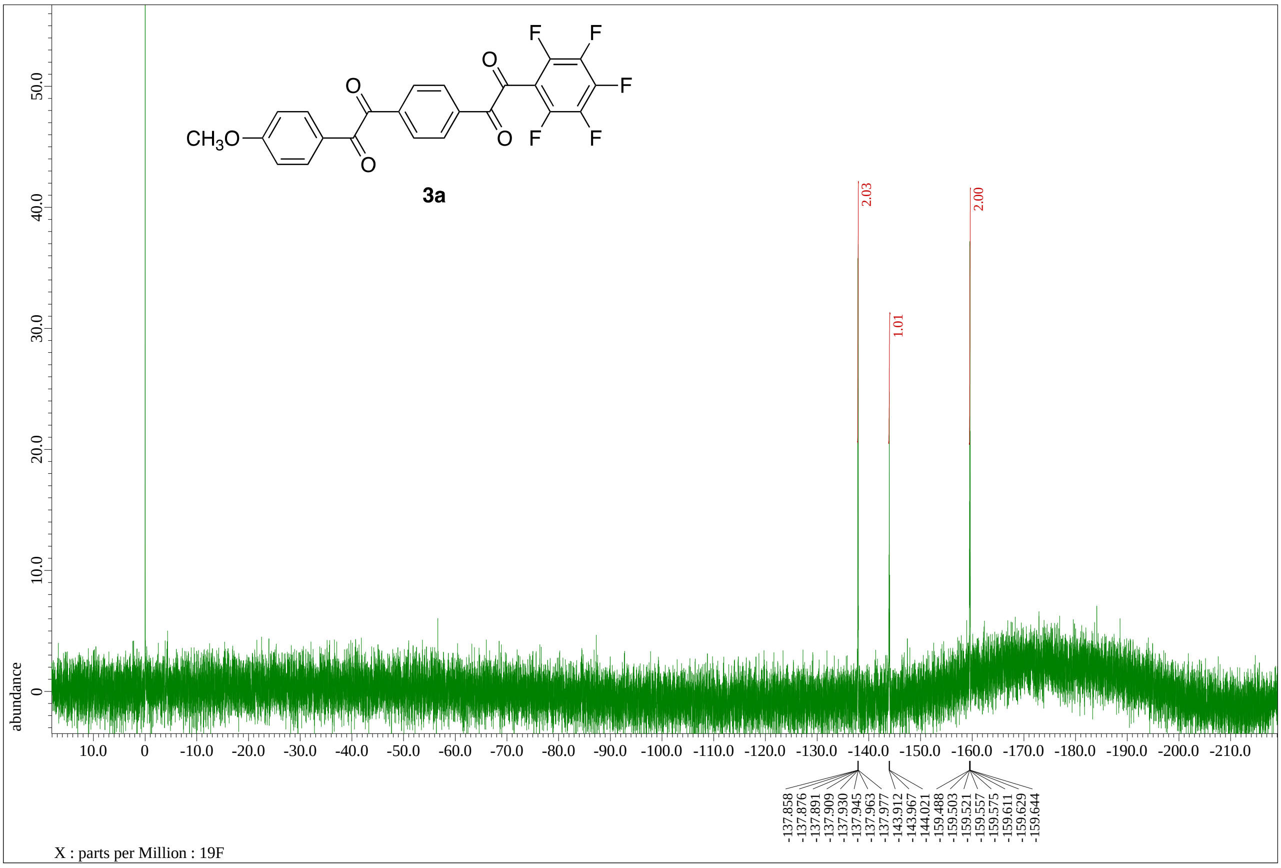

^{1}H NMR spectrum for **3b** ($CDCl_3$, 400 MHz)

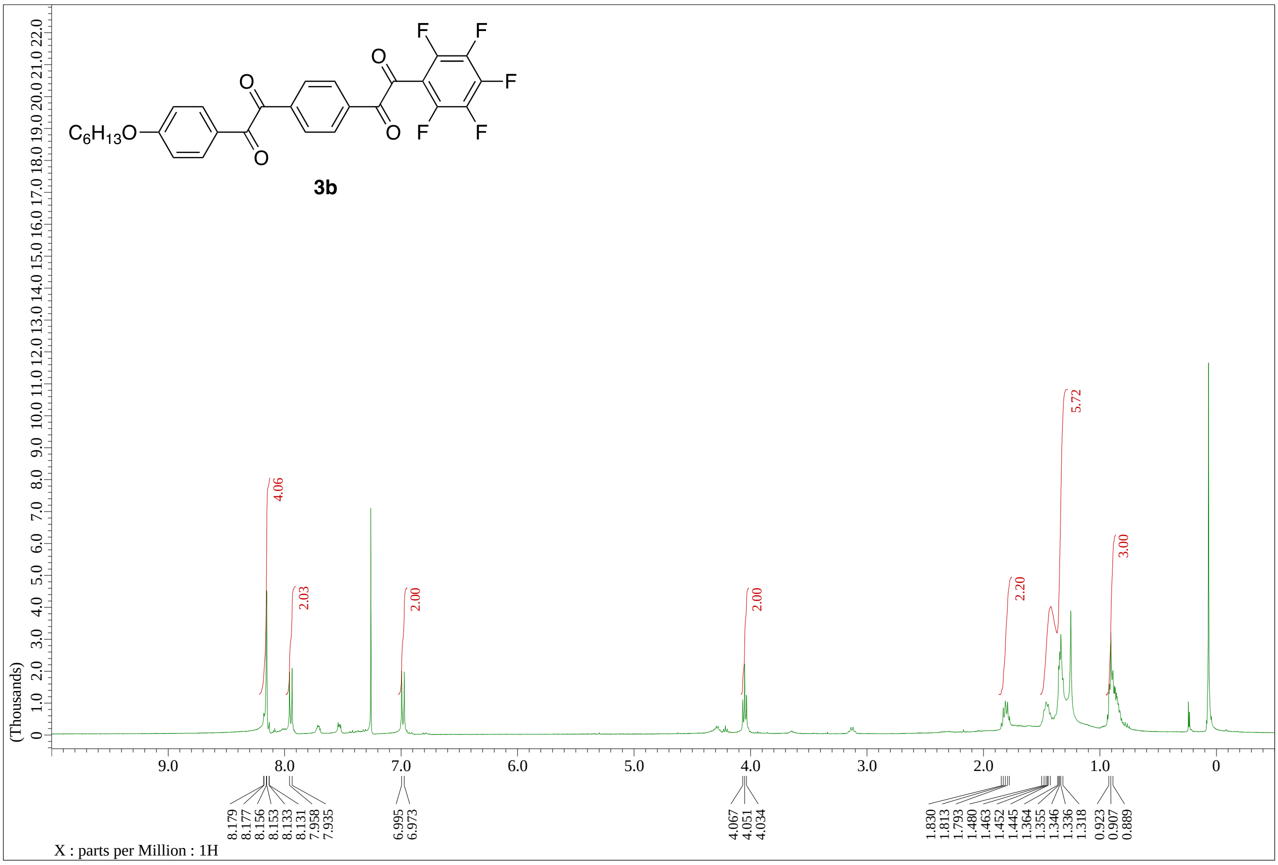

^{13}C NMR spectrum for **3b** ($CDCl_3$, 100 MHz)

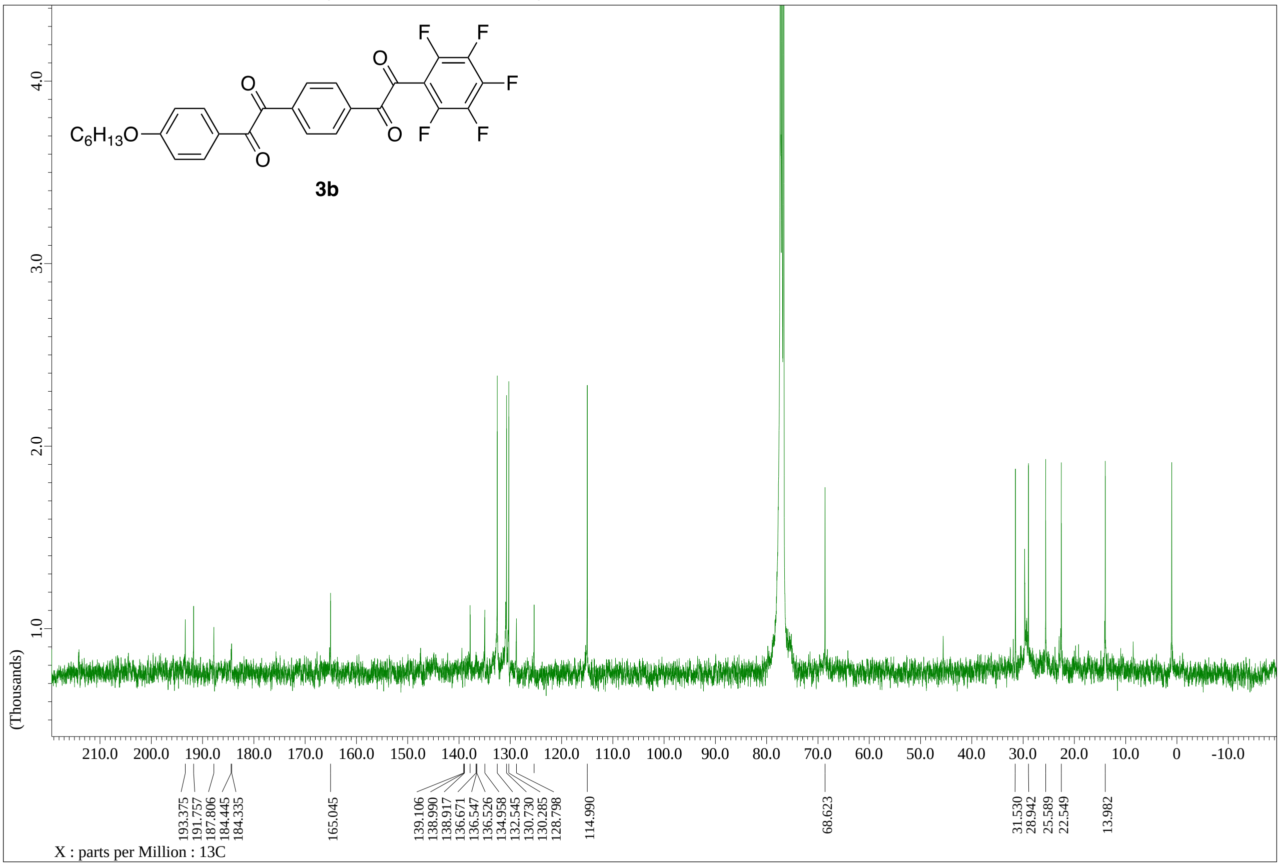

^{19}F NMR spectrum for **3b** ($CDCl_3$, 376 MHz)

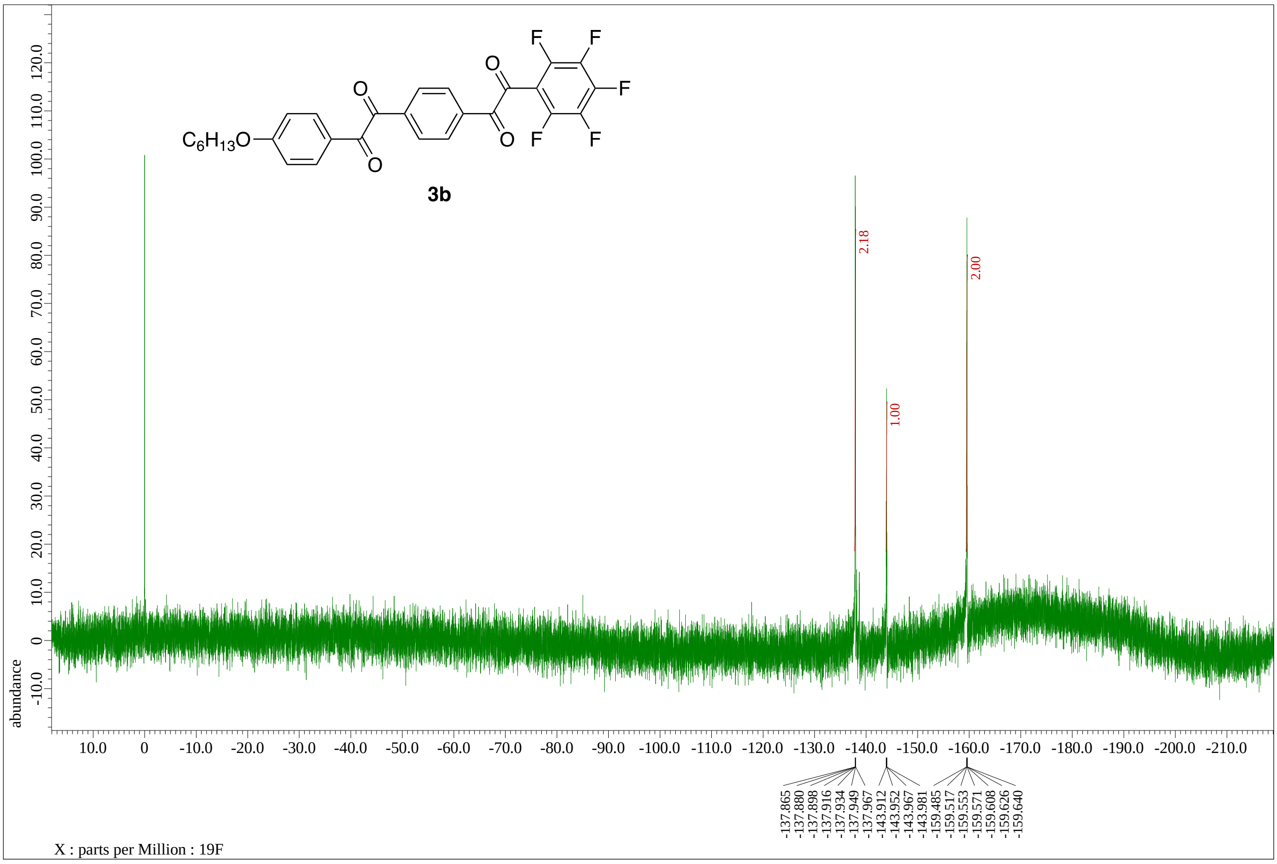

^{1}H NMR spectrum for **3c** ($CDCl_3$, 400 MHz)

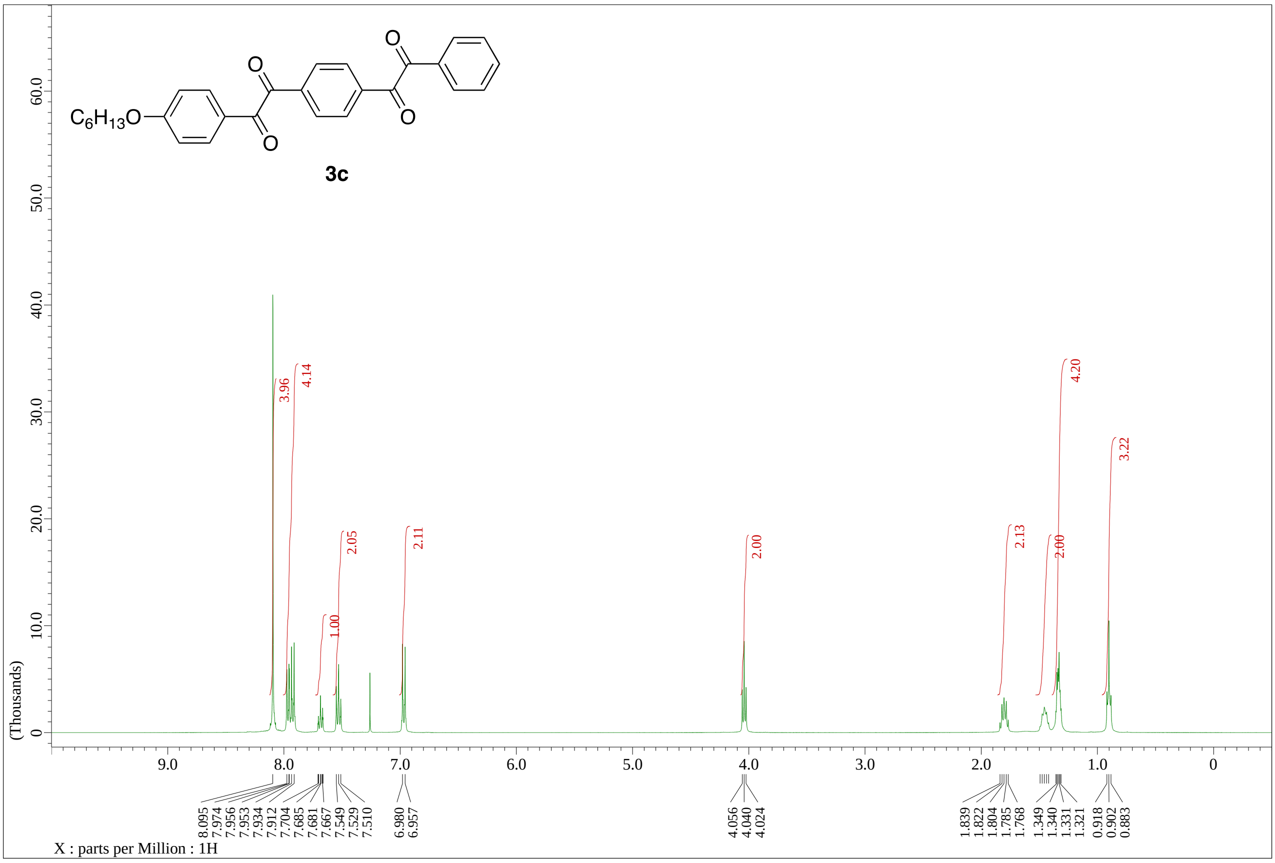

^{13}C NMR spectrum for **3c** ($CDCl_3$, 100 MHz)

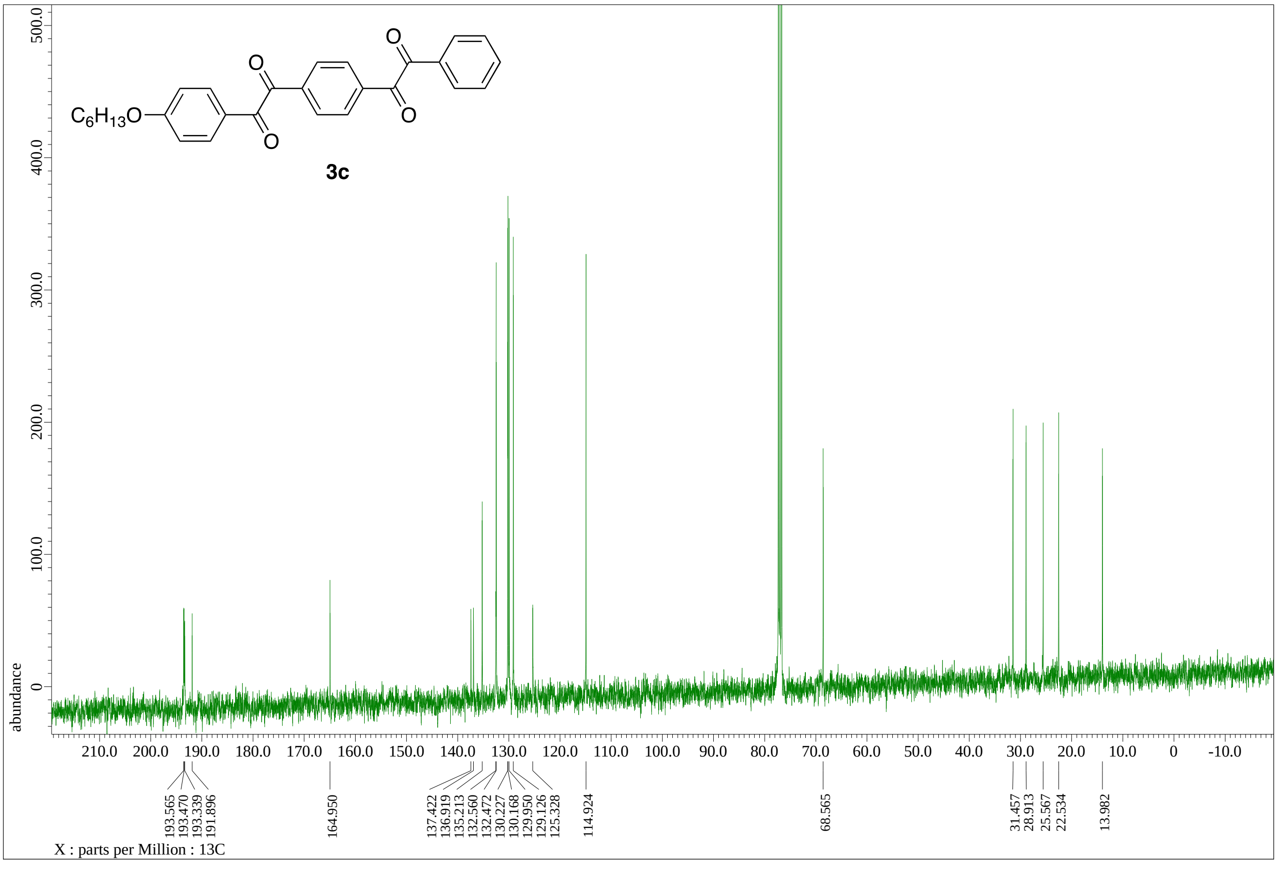

3. Computation

All computations were performed by a density functional theory (DFT) using the Gaussian 16 (Rev. B.01) suite of programs. Geometry optimizations were executed at the CAM-B3LYP/6-31G(d) level of theory with the implicit solvation model, namely, the conductor-like polarizable continuum model (CPCM), for heptane. The vertical electronic transitions were calculated using a time-dependent (TD)-DFT method at the same level of theory.

3-1. Optimized structure (charge distribution) of 1a

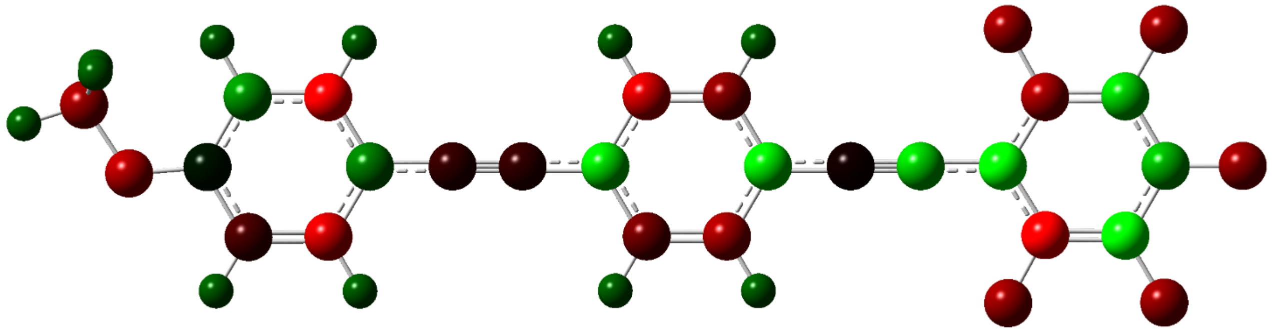

SCF Done: E (RCAM-B3LYP) = –1456.76520762 hartree

Dipole moment (field-independent basis, Debye-Ang):

X = –4.9568, Y = 1.2690, Z = 0.0001, Tot = 5.1167

3-2. Cartesian coordinates

No.	Atom No.	Type	Coordinates (Angstroms) x	y	z
1	6	0	-11.09	0.873484	0.000009
2	1	0	-10.9045	1.47744	0.895991
3	1	0	-12.1259	0.534657	-0.000058
4	1	0	-10.9044	1.47761	-0.895852
5	8	0	-10.299	-0.299563	-0.000073
6	6	0	-8.94753	-0.173342	-0.000049
7	6	0	-8.22376	-1.37095	-0.000137
8	1	0	-8.77109	-2.30767	-0.000225
9	6	0	-6.84285	-1.34824	-0.000111
10	1	0	-6.28671	-2.28009	-0.000185
11	6	0	-6.14231	-0.129029	0.000003
12	6	0	-6.8783	1.05909	0.0001
13	1	0	-6.35331	2.00894	0.000194
14	6	0	-8.26954	1.04603	0.000075
15	1	0	-8.80717	1.98632	0.000159
16	6	0	-4.71431	-0.107056	0.000031
17	6	0	-3.50376	-0.091643	0.000049
18	6	0	-1.3467	-1.27198	-0.000102
19	1	0	-1.87907	-2.21724	-0.000211
20	6	0	0.037781	-1.25508	-0.000108
21	1	0	0.594707	-2.1863	-0.000217
22	6	0	0.73295	-0.037224	0.000029
23	6	0	0.006753	1.1624	0.000186
24	1	0	0.539664	2.10758	0.000301
25	6	0	-1.37767	1.14371	0.000192
26	1	0	-1.93413	2.07501	0.000314
27	6	0	-2.07656	-0.073287	0.000041
28	6	0	2.15988	-0.018659	0.000017
29	6	0	3.36832	-0.002205	0.000013
30	6	0	4.78789	0.017019	0.000002
31	6	0	5.53557	-1.16517	0.000052
32	6	0	6.92003	-1.15512	0.000042
33	6	0	7.59898	0.05526	-0.000027
34	6	0	6.88737	1.24673	-0.000074
35	6	0	5.50315	1.2191	-0.000064
36	9	0	4.90939	-2.34385	0.000118
37	9	0	7.60473	-2.30023	0.000093
38	9	0	8.93022	0.073351	-0.000037
39	9	0	7.5407	2.41005	-0.000135
40	9	0	4.84515	2.38037	-0.000112

3-3. Optimized structure (charge distribution) of 1c

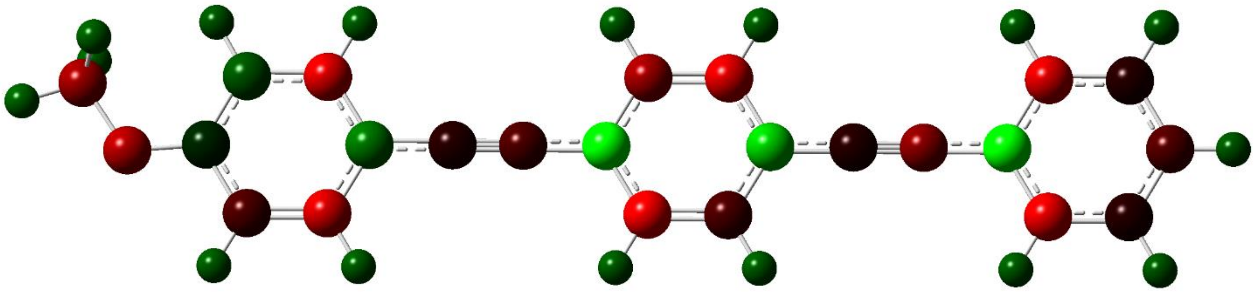

SCF Done: E (RCAM-B3LYP) = –960.681573201 hartree

Dipole moment (field-independent basis, Debye-Ang):

X = 1.2195, Y = –1.3069, Z = 0.0007, Tot = 1.7875

3-4. Cartesian coordinates

No.	Atom No.	Type	Coordinates (Angstroms) x	y	z
1	6	0	9.42305	-0.861537	0.000486
2	1	0	9.23864	-1.46564	0.896729
3	1	0	10.4586	-0.521509	0.00005
4	1	0	9.23827	-1.46682	-0.894881
5	8	0	8.63057	0.309972	-0.000127
6	6	0	7.27837	0.180508	-0.000069
7	6	0	6.55168	1.376	-0.001034
8	1	0	7.09649	2.31424	-0.001769
9	6	0	5.17054	1.34986	-0.001039
10	1	0	4.6122	2.2804	-0.001799
11	6	0	4.47232	0.129399	-0.000084
12	6	0	5.21173	-1.0565	0.000869
13	1	0	4.6891	-2.00766	0.001617
14	6	0	6.60324	-1.04012	0.000887
15	1	0	7.14291	-1.97929	0.001664
16	6	0	3.04395	0.104069	-0.000089
17	6	0	1.83344	0.085372	-0.000087
18	6	0	-0.328792	1.25857	-0.000206
19	1	0	0.200247	2.20592	-0.000333
20	6	0	-1.71362	1.237	-0.000185
21	1	0	-2.27185	2.1675	-0.000296
22	6	0	-2.41004	0.019411	-0.000021
23	6	0	-1.67617	-1.17598	0.000119
24	1	0	-2.20529	-2.12334	0.000252
25	6	0	-0.291357	-1.15448	0.0001
26	1	0	0.266841	-2.08498	0.000207
27	6	0	0.405539	0.063136	-0.000065
28	6	0	-3.83837	-0.002775	-0.000003
29	6	0	-5.04852	-0.02163	0.000011
30	6	0	-6.4785	-0.043963	0.000028
31	6	0	-7.20843	1.15328	0.001269
32	6	0	-8.5972	1.12791	0.001289
33	6	0	-9.27725	-0.087711	0.000071
34	6	0	-8.55955	-1.28148	-0.001169
35	6	0	-7.17066	-1.26342	-0.001195
36	1	0	-6.67571	2.09878	0.002219
37	1	0	-9.15149	2.06177	0.002261
38	1	0	-10.363	-0.104668	0.000089
39	1	0	-9.08437	-2.23221	-0.002122
40	1	0	-6.60867	-2.19182	-0.002169

3-5. Optimized geometry of 2a

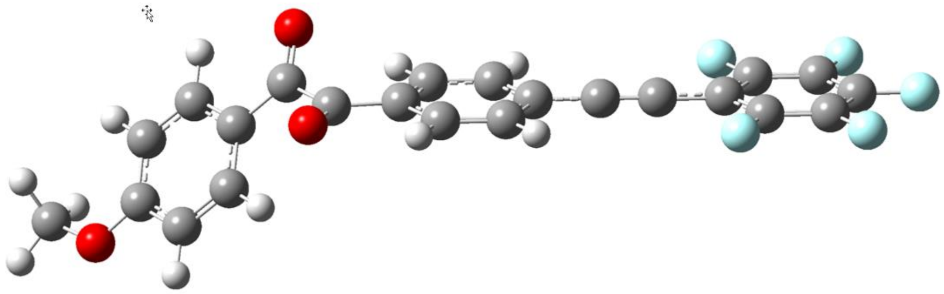

SCF Done: E (RCAM-B3LYP) = –1607.22472657 hartree

Dipole moment (field-independent basis, Debye-Ang):

X = –3.5454, Y = 2.5640, Z = –1.8632, Tot = 4.7556

3-6. Cartesian coordinates of 2a

No.	Atom No.	Type	Coordinates (Angstroms) x	y	z
1	6	0	-7.79207	0.664165	-0.994869
2	6	0	-6.90737	-0.291934	-1.50785
3	6	0	-5.78984	-0.660464	-0.784976
4	6	0	-5.5279	-0.081481	0.467166
5	6	0	-6.41794	0.872884	0.966864
6	6	0	-7.54476	1.24949	0.251881
7	1	0	-7.12504	-0.733508	-2.47431
8	1	0	-5.12542	-1.41617	-1.18747
9	1	0	-6.21086	1.31903	1.93411
10	1	0	-8.21739	1.9904	0.666081
11	6	0	-4.33521	-0.407147	1.26976
12	6	0	-3.36131	-1.48477	0.775139
13	6	0	-1.91294	-1.16366	0.666698
14	6	0	-1.04407	-2.18769	0.27329
15	6	0	-1.40528	0.116985	0.90568
16	6	0	0.308851	-1.94175	0.122795
17	1	0	-1.45256	-3.17572	0.089802
18	6	0	-0.050233	0.370268	0.752844
19	1	0	-2.06331	0.91447	1.2293
20	6	0	0.819222	-0.655787	0.360874
21	1	0	0.983298	-2.73583	-0.179698
22	1	0	0.345439	1.36266	0.940614
23	8	0	-4.0912	0.107549	2.34942
24	8	0	-3.80291	-2.59381	0.53562
25	8	0	-8.85667	0.957871	-1.77382
26	6	0	-9.79951	1.91235	-1.31661
27	1	0	-9.33225	2.89364	-1.17722
28	1	0	-10.5567	1.97812	-2.0974
29	1	0	-10.2673	1.58831	-0.380287
30	6	0	2.21457	-0.395005	0.205293
31	6	0	3.39485	-0.173679	0.074391
32	6	0	4.78163	0.092074	-0.077609
33	6	0	5.30998	1.36543	0.158716
34	6	0	5.67901	-0.906616	-0.469528
35	6	0	6.66031	1.63423	0.013795
36	6	0	7.03212	-0.65435	-0.619128
37	6	0	7.52435	0.620533	-0.376503
38	9	0	4.50128	2.35751	0.534676
39	9	0	7.13536	2.8585	0.246275
40	9	0	8.82336	0.870681	-0.517928
41	9	0	7.86441	-1.62652	-0.994148
42	9	0	5.23221	-2.14048	-0.709095

3-7. Optimized geometry of 3a

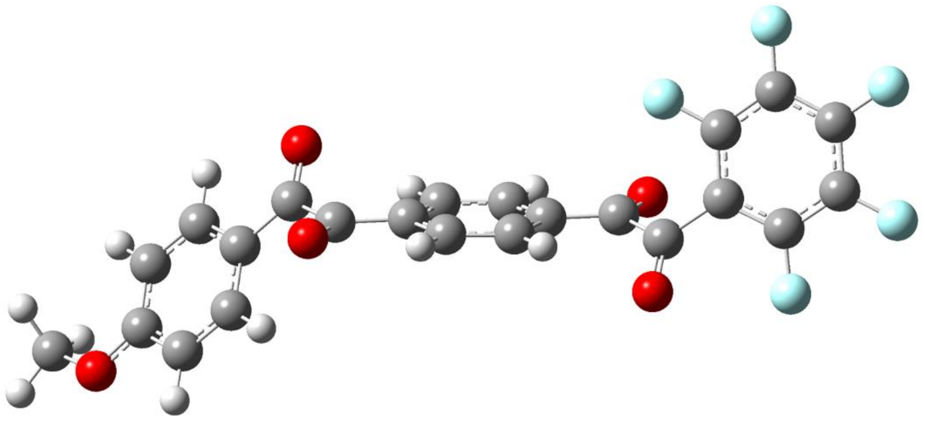

SCF Done: E (RCAM-B3LYP) = –1757.67288894 hartree

Dipole moment (field-independent basis, Debye-Ang):

X = –4.5783, Y = –0.3793, Z = –1.3769, Tot = 4.7959

3-8. Cartesian coordinates of 3a

No.	Atom No.	Type	Coordinates (Angstroms) x	y	z
1	6	0	-8.1024	-0.641623	-0.29745
2	6	0	-7.12931	-1.33762	0.429992
3	6	0	-5.90044	-0.757427	0.67426
4	6	0	-5.61212	0.530561	0.194632
5	6	0	-6.59058	1.21297	-0.533514
6	6	0	-7.82969	0.642788	-0.781878
7	1	0	-7.36778	-2.33001	0.796913
8	1	0	-5.16553	-1.30207	1.25561
9	1	0	-6.36287	2.20704	-0.904306
10	1	0	-8.56937	1.19672	-1.34654
11	6	0	-4.30934	1.18695	0.394667
12	6	0	-3.23841	0.501852	1.2536
13	6	0	-1.86361	0.318343	0.700827
14	6	0	-0.893374	-0.228165	1.54437
15	6	0	-1.53696	0.634569	-0.622263
16	6	0	0.391148	-0.459862	1.07781
17	1	0	-1.16568	-0.470715	2.56578
18	6	0	-0.252552	0.404761	-1.08986
19	1	0	-2.27685	1.07672	-1.27853
20	6	0	0.717376	-0.143974	-0.245947
21	1	0	1.1301	-0.896578	1.73862
22	1	0	0.019385	0.652454	-2.11013
23	6	0	2.08139	-0.331576	-0.796345
24	6	0	3.15365	-1.07878	0.015177
25	6	0	4.54114	-0.523154	-0.029653
26	6	0	5.65414	-1.35575	-0.146371
27	6	0	4.77192	0.847111	0.063814
28	6	0	6.94097	-0.840206	-0.182485
29	6	0	6.04774	1.38207	0.043413
30	6	0	7.13633	0.530059	-0.083177
31	8	0	-4.03344	2.29066	-0.047694
32	8	0	-3.52549	0.180274	2.39061
33	8	0	2.89217	-2.13207	0.548802
34	8	0	2.40816	0.011586	-1.91504
35	9	0	3.73844	1.68348	0.214222
36	9	0	6.23839	2.69582	0.154553
37	9	0	8.36723	1.02785	-0.105861
38	9	0	7.99014	-1.65078	-0.313828
39	9	0	5.51022	-2.67207	-0.268834
40	8	0	-9.2733	-1.28958	-0.481151
41	6	0	-10.3126	-0.642756	-1.19633
42	1	0	-10.0064	-0.422147	-2.22499
43	1	0	-11.1454	-1.34526	-1.20794
44	1	0	-10.6206	0.281333	-0.694747
